# Fluorinated Naphthalene Diimides as Buried Electron Transport Materials Achieve Over 23% Efficient Perovskite Solar Cells

**DOI:** 10.1002/advs.202403735

**Published:** 2024-07-23

**Authors:** Xiaofeng Li, Wanhai Wang, Pengyu Huang, Li Yang, Jianfei Hu, Kun Wei, Liang Gao, Yonghe Jiang, Kexuan Sun, Guozheng Du, Xuanyi Cai, Chang Liu, Weihua Tang, Jinbao Zhang

**Affiliations:** ^1^ College of Materials Fujian Key Laboratory of Advanced Materials Xiamen Key Laboratory of Electronic Ceramic Materials and Devices Xiamen University Xiamen 361005 China; ^2^ Institute of Flexible Electronics (IFE, Future Technologies) College of Materials Innovation Laboratory for Sciences and Technologies of Energy Materials of Fujian Province (IKKEM) Xiamen University Xiamen 361102 China; ^3^ School of Chemistry and Chemical Engineering Nanjing University of Science and Technology Nanjing 210094 China; ^4^ Shenzhen Research Institute of Xiamen University Shenzhen 518000 China; ^5^ Zhejiang Provincial Engineering Research Center of Energy Optoelectronic Materials and Devices Ningbo Institute of Materials Technology & Engineering Chinese Academy of Sciences Ningbo 315201 China

**Keywords:** electron transport materials, fluorinated naphthalene diimide, high efficiency, n‐i‐p perovskite solar cells, stability

## Abstract

Naphthalene diimides (NDI) are widely serving as the skeleton to construct electron transport materials (ETMs) for optoelectronic devices. However, most of the reported NDI‐based ETMs suffer from poor interfaces with the perovskite which deteriorates the carrier extraction and device stability. Here, a representative design concept for editing the peripheral groups of NDI molecules to achieve multifunctional properties is introduced. The resulting molecule 2,7‐bis(2,2,3,3,4,4,4‐heptafluorobutyl)benzo[*lmn*][3,8]phenanthroline‐1,3,6,8(2*H*,7*H*)‐tetraone (NDI‐C4F) incorporated with hydrophobic fluorine units contributes to the prevention of excessive molecular aggregation, the improvement of surface wettability and the formation of strong chemical coordination with perovskite precursors. All these features favor retarding the perovskite crystallization and achieving superior buried interfaces, which subsequently promote charge collection and improve the structural compatibility between perovskite and ETMs. The corresponding PSCs based on low‐temperature processed NDI‐C4F yield a record efficiency of 23.21%, which is the highest reported value for organic ETMs in n‐i‐p PSCs. More encouragingly, the unencapsulated devices with NDI‐C4F demonstrate extraordinary stability by retaining over 90% of their initial PCEs after 2600 h in air. This work provides an alternative molecular strategy to engineer the buried interfaces and can trigger further development of organic ETMs toward reliable PSCs.

## Graphical abstract

A representative design concept for editing the peripheral groups of naphthalene diimides (NDI) molecules is proposed. The fluorinate NDI (NDI‐C4F) contributes to the prevention of excessive molecular aggregation, the improvement of surface wettability, and the formation of strong chemical coordination with perovskite precursors.

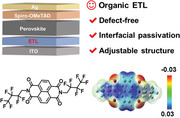

## 1 Introduction

Perovskite solar cells (PSCs) have emerged as promising candidates for affordable photovoltaic technologies because of unprecedented power conversion efficiencies (PCEs) and simple manufacturing processes. Aided by the technological advances in material development,^[^ ^1^, ^2^ ^]^ and device engineering,^[^ ^3^, ^4^ ^]^ single‐junction PSCs have achieved record PCEs exceeding 26%,^[^ ^5^ ^]^ even rivaling the performance of commercial silicon solar cells. In the sandwich‐structured PSCs, charge transport materials and their interfaces with the perovskite play critical roles in device efficiency and stability. Specifically, electron transport layers (ETLs) are located underneath the perovskite layer in regular n‐i‐p architectures, which significantly influences the charge‐extraction efficiency,^[^ ^6^, ^7^, ^8^ ^]^ impacts perovskite crystallization kinetics, and affects the perovskite film quality.^[^ ^9^ ^]^

To date, SnO_2_ prevails as the state‐of‐the‐art ETL for n‐i‐p devices due to its favorable energy alignment, high transparency, and superior electron mobility.^[^ ^10^ ^]^ In spite of high performance, SnO_2_ suffers from several disadvantages limiting its scalable application. For example, SnO_2_ films based on nanocolloidal particles are prone to surface defects and low‐crystallinity nature.^[^ ^11^ ^]^ The self‐doped hydroxyl groups (–OH) and dangling bonds negatively impact the structural and electronic properties of SnO_2_, thereby causing the deterioration of the interfaces and the operational stabilities of PSCs.^[^ ^12^ ^]^ The surface defects on SnO_2_ alter the heterogeneous nucleation of perovskite, leading to the formation of structural defects at the perovskite‐buried interfaces. These defects subsequently behave as an initiators of carrier recombination losses and interfacial degradation under heat or light stimulation. To circumvent these issues, many efforts have been carried out to adjust SnO_2_ properties through additive strategies or surface passivation.^[^ ^13^, ^14^ ^]^ However, the reported approaches either increase the complexity of manufacturing or induce barriers inhibiting the carrier extraction at the interfaces.

**Figure 1:**
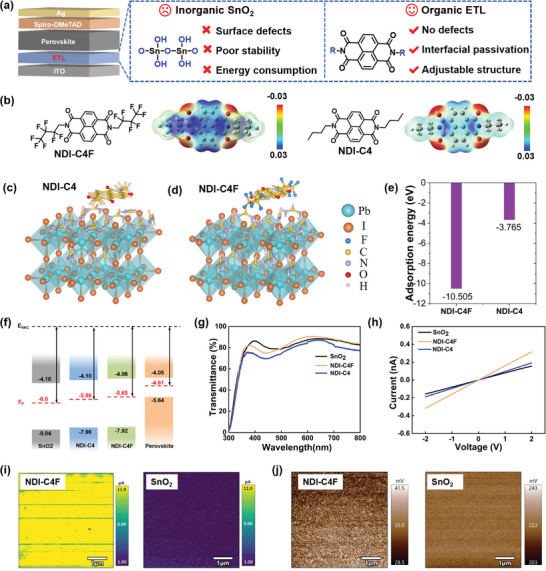
a) Properties of inorganic SnO_2_ and organic ETMs in n‐i‐p PSCs. b) Molecular structures and electrostatic surface potential (ESP) of NDI‐C4F and NDI‐C4. c–e) Simulated adsorption energy between the molecules and the perovskite. f) Energy‐level diagram of ETLs and perovskite. g) UV–vis transmittance spectroscopy and h) conductivity of different ETLs. i) Conductive atomic force microscopy (C‐AFM) and j) Surface kelvin probe force microscope (KPFM) images of the film based on NDI‐C4F and SnO_2_.

Alternatively, organic molecules as ETLs for PSCs show high promises due to their low‐temperature fabrication, chemical versatility, and defect‐free properties (**Figure** **1**a). In addition, the structural tunability of organic molecules renders them an adaptable platform for functionalization, which could provide targeted interaction with the perovskites to realize effective defect passivation.^[^ ^15^ ^]^ The reported organic ETLs are mostly composed of naphthalene diimides (NDIs) skeleton because of superior electron‐transport capability and suitable energy levels.^[^ ^16^ ^]^ Meanwhile, the carbonyl groups in NDIs are able to chelate with non‐coordinated Pb^2+^ to passivate the defects on the surface of perovskites.^[^ ^17^ ^]^ Despite these benign properties, the large π‐conjugated NDI is usually accompanied by excessive molecular aggregation, manifesting as low film‐forming ability and morphological variations.^[^ ^18^ ^]^ The poor surface wettability of NDI‐based ETLs is another challenging factor limiting the large‐scale perovskite deposition. These drawbacks hinder the application of organic ETLs in n‐i‐p PSCs. To the best of our knowledge, the PCEs of n‐i‐p PSCs with organic ETLs (<20.0%) lag far behind those of their inorganic counterparts.^[^ ^19^ ^]^ Therefore, the development of intrinsically stable and efficient organic ETLs for n‐i‐p devices through comprehensive molecular design strategies is highly desired.

As learned from the general molecular engineering of organic semiconductors, the introduction of side chains to the conjugated skeleton is conducive to preventing excessive aggregation of molecules, thereby optimizing the crystallinity and film morphology of ETLs.^[^ ^20^ ^]^ For reducing the interfacial carrier recombination caused by defects and trap states, functional groups containing heteroatoms are usually incorporated into the molecules to serve as the interaction sites.^[^ ^21^ ^]^ For improving the surface wettability, affinity groups for perovskite precursor solutions are also required. In these regards, delicate fluorinated compounds show high promise in triggering the chemical interaction with perovskite cations for defects passivation and ion immobilization.^[^ ^22^ ^]^ Moreover, the astonishing hydrophobicity and thermal‐resistant properties of fluorinated molecules have shown positive effects on improving the operational stability of PSCs.^[^ ^23^ ^]^

In this work, we design and synthesize an organic ETM, namely 2,7‐bis(2,2,3,3,4,4,4‐heptafluorobutyl)benzo[*lmn*][3,8]phenanthroline‐1,3,6,8(2*H*,7*H*)tetraone (NDI‐C4F) for n‐i‐p PSCs. The incorporation of hydrophobic fluorine units in NDI forms chemical interaction with the perovskite precursors for precisely controlling the perovskite nucleation processes, which subsequently improve the perovskite crystallinity and enhance the morphological uniformity of the buried interfaces. The NDI‐C4F with fluorine side‐group substitution initiates superior electrical interaction with the perovskite film, which favors accelerating the charge separation and inhibiting the carrier recombination losses. Consequently, the NDI‐C4F as the ETM in PSCs yields a remarkable PCE of 23.21%, which represents the highest reported efficiency for organic ETMs in n‐i‐p devices. Furthermore, the unsealed devices with NDI‐C4F demonstrate extraordinary environmental stability by retaining over 90% of their initial PCEs after 2600 h of storage in the air condition. More importantly, NDI‐C4F has been generally applicable for different perovskites in both flexible and large‐area devices. This study reveals that molecular functionalization of organic ETMs is essential for engineering the buried interfaces and increasing the photovoltaic performance and would provide a new avenue for the design of efficient and stable organic ETMs for n‐i‐p PSCs.

## 2 Results and Discussion

### 2.1 SAM Properties

On the basis of the NDI units, the fluorinated alkyl side chains have been incorporated into the structure of NDI‐C4F, as illustrated in Figure 1b. The as‐designed NDI‐C4F was synthesized by a low‐cost, one‐step reaction, and facile purification method, as provided in the Supporting Information (Note S1, Figures S1 and S2, Supporting Information). Based on the rough estimation, the synthetic cost of NDI‐C4F was ≈49.6 $ per gram (Table S1, Supporting Information), which is affordable for large‐scale applications. For the purpose of investigating the critical roles of fluorine substitution, the unfluorinated counterpart 2,7‐dibutylbenzo[*lmn*][3,8]phenanthroline‐1,3,6,8(2*H*,7*H*)‐tetraone (NDI‐C4) was also prepared for reference (Figure 1b; Figures S3 and S4, Supporting Information).

To study the intrinsic optoelectronic characteristics of NDI‐C4F, the electron density distribution of molecular ETMs was examined by visualizing the electrostatic surface potential (ESP) in the molecules.^[^ ^24^ ^]^ The carbonyl groups of NDIs in two molecules present high negative charge delocalization (red), which may function as Lewis bases to interact with under‐coordinated Pb^2+^ cations.^[^ ^25^ ^]^

To better understand the interacting mechanism between the molecules and perovskite, we have simulated their chemical interaction by DFT. As shown in Figure 1c–e, the incorporation of F atoms in NDI‐C4F could contribute much higher adsorption energy with FA and I in the perovskite by forming hydrogen bonds. We also investigated the energetic alignment between these molecules and the perovskite by ultraviolet photoelectron spectroscopy (UPS) (Figure 1f; Figure S5, Supporting Information). The lowest unoccupied molecular orbital (LUMO) of SnO_2_, NDI‐C4, and NDI‐C4F on ITO were calculated to be −6.0, −5.86, and −5.65 eV, respectively. Based on the energetic values of perovskite,^[^ ^26^ ^]^ NDI‐C4F showed the smallest energy offset with the perovskite compared to SnO_2_ and NDI‐C4, which favors reducing the energy losses during the carrier extraction at the interfaces. Considering that light transmittance of ETLs impacts the light absorption capacity and the short‐circuit current (*J_sc_*) in the devices, UV–vis transmittance spectroscopy was conducted (Figure 1g). The film based on NDI‐C4F showed a higher transmittance compared to NDI‐C4 and SnO_2_, especially in the range of 500–800 nm, which suggests that NDI‐C4F as ETLs could contribute to stronger light harvesting and higher photocurrent in PSCs.^[^ ^27^ ^]^

Charge conductivity of ETLs were investigated by four‐point probe measurement (Figure 1h). Obviously, a much lower resistivity was obtained for NDI‐C4F‐based film compared to that for NDI‐C4 and SnO_2_, which is beneficial for electron‐collection processes in the devices. According to the conductive atomic force microscopy (C‐AFM) (Figure 1i), NDI‐C4F film exhibited a much higher and more uniform spatial current relative to SnO_2_. Based on the surface Kelvin probe force microscope (KPFM) (Figure 1j), the NDI‐C4F film showed a lower surface potential difference of 0.035 V compared with the SnO_2_ film (0.223 V), suggesting a lower energy barrier for the electron extraction at the ETL/perovskite interface.

### 2.2 Effects on Perovskite Films

**Figure 2:**
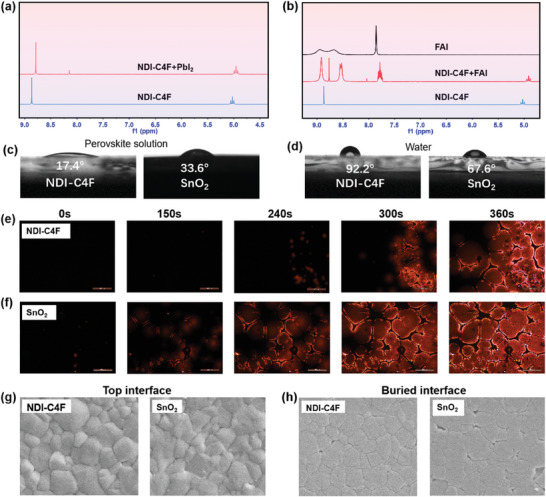
^1^H NMR spectra of NDI‐C4F when mixed with a) PbI_2_ and b) FAI. Contact angles of different ETL films with c) the perovskite precursor solution and d) water. Fluorescence polarizing microscope images of perovskite films coated on the e) NDI‐C4F and f) SnO_2_. The SEM images of g) the top surface and h) the buried interface of the perovskite films prepared on NDI‐C4F and SnO_2_.

As the crystallization template of the perovskite layer, ETLs exert critical roles in the perovskite growth and film quality. To elucidate the interaction mechanism, proton nuclear magnetic resonance (^1^H NMR) was conducted.^[^ ^28^, ^29^ ^]^ As shown in **Figure** **2**a, evident down‐field shifts of proton peaks were observed in the NDI‐C4F sample after mixing with PbI_2_, suggesting the chemical coordination interactions between NDI‐C4F and the Pb^2+^ ions. Furthermore, the interaction between formamidinium iodide (FAI) and NDI‐C4F was simulated (Figure 2b). Similarly, apparent down‐field shifts and clearly split peaks (at 8.9, 8.5, and 7.8 ppm) were observed after mixing these two molecules. These results demonstrate that NDI‐C4F initiates multiple interactions with the perovskite precursors, which could induce significant impacts on the crystallization kinetics of the liquid precursors as well as defect passivation to the solid films.^[^ ^30^ ^]^ Besides, NDI‐C4F could form chemical adsorption on the ITO due to chemical coordination between In^3+^ and fluorine groups, as confirmed by the X‐ray photoelectron spectroscopy (XPS) where the binding energy for In 3d signal from ITO was shifted from 443.97 to 444.55 eV after coated with NDI‐C4F (Figure S6, Supporting Information).

The surface properties of ETLs, such as wettability and roughness, are crucial factors influencing the perovskite microstructures and interfacial electronic properties.^[^ ^31^ ^]^ Accordingly, we measured the contact angles (CAs) of different ETLs with the perovskite solution or water (Figure 2c). NDI‐C4F showed a smaller CA with the perovskite precursor (17.4°) compared to SnO_2_ (33.6°) and NDI‐C4 (66.2°) (Figure S7, Supporting Information). The smaller contact angle observed of NDI‐C4F and the perovskite precursor is due to strong interactions between NDI‐C4F and FAI, as well as Pb^2+^ in the perovskite, which allows the perovskite to distribute more uniformly across the organic layer. In the case of CA with water, NDI‐C4F exhibited the highest hydrophobicity (Figure 2d). This implies that the introduction of fluorines in the ETLs effectively enhances the contact with perovskite and repels the water from the environment.^[^ ^32^ ^]^ As a result, the formed polycrystalline perovskite films on the basis of NDI‐C4F exhibited a smooth surface (RMS = 2.89 nm) as compared to that for SnO_2_ (RMS = 4.02 nm), as examined by atomic force microscopy (AFM) (Figure S8, Supporting Information).

The surface properties of NDI‐C4F and the interaction with the perovskite precursors are expected to affect the nucleation and growth of the perovskite.^[^ ^33^, ^34^ ^]^ The perovskite formation on different ETLs was monitored by a fluorescence polarizing microscope, as illustrated in Figure 2e,f. The perovskite nucleation rate was significantly retarded on the ETL based on NDI‐C4F compared to that for SnO_2_ counterparts, which resulted in enlarged grain sizes and improved crystallinity, as revealed by top‐view scanning electron microscopy (SEM) images (Figure 2g). To visualize the microstructures of the perovskite buried interfaces, a nondestructive technique was implemented to exfoliate the entire perovskite films from the substrates (Figure S9, Supporting Information).^[^ ^35^ ^]^ The perovskite grown on the NDI‐C4F showed impressive film uniformity and high‐quality grains as compared to poor buried interfaces on the SnO_2_ where large voids and pinholes were observed (Figure 2h). The superior interfaces between NDI‐C4F‐based ETLs and the perovskite build the foundation for sufficient charge extraction and superior interfacial stability of the devices.

**Figure 3:**
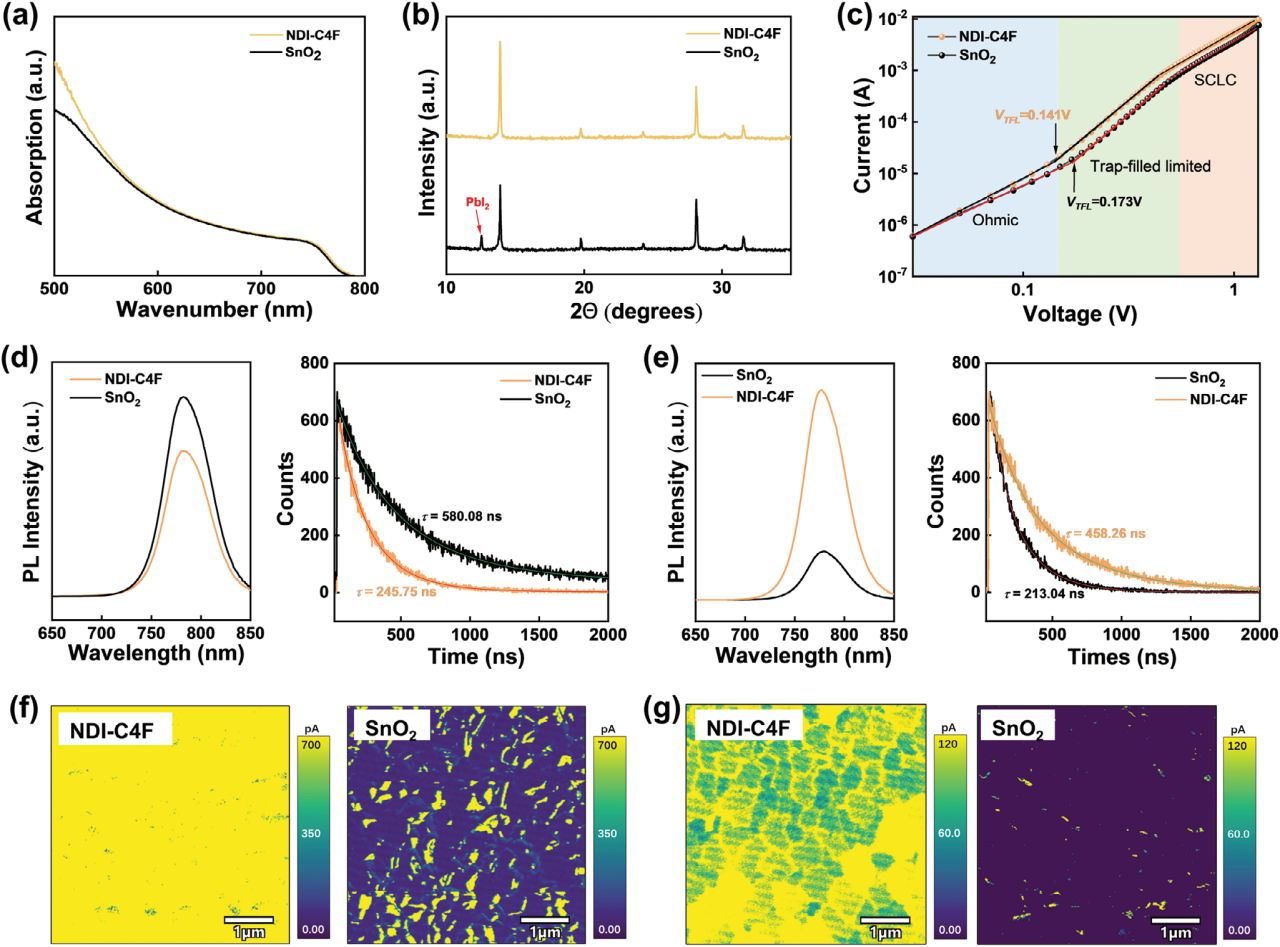
a) UV–vis absorption spectra and b) XRD patterns of the perovskite films coated on NDI‐C4F and SnO_2_. c) Dark *I–V* curves of the electron‐only devices based on NDI‐C4F and SnO_2_. d) The PL and TRPL spectra of the sample based on ITO/ETL/perovskites and e) the exfoliated perovskite from the substrate. f) The C‐AFM mapping of the perovskite surface and g) the buried interfaces after exfoliation.

To further explore the impacts of ETLs on the microscopic properties of the perovskite films, UV–vis absorption and X‐ray diffraction (XRD) of the corresponding perovskite layers were performed (**Figure** **3**a). Due to the positive roles of NDI‐C4F on the formation of perovskite, a much stronger light absorption accompanied by higher crystallinity was obtained in the perovskite films based on NDI‐C4F compared to that on the SnO_2_, which would be beneficial for obtaining higher photovoltaic parameters in the devices (Figure 3b).^[^ ^36^ ^]^ According to the space charge limited current (SCLC) (Figure 3c), the trap filling limit voltage (*V_TFL_*) and the electron defect density (*N_t_*) can be estimated.^[^ ^37^ ^]^ The devices based on NDI‐C4F exhibited a much smaller *V*_TFL_ (0.141 V) and a lower *N_t_* (3.79 × 10^16^ cm^−3^) compared to those with the SnO_2_ (*V*_TFL_ =  0.173 V and *N_t_* =  4.65 × 10^16^ cm^−3^), which is expected to mitigate the non‐radiative recombination losses in the devices.

Steady‐state photoluminescence (PL) and time‐resolved photoluminescence (TRPL) (Note S2, Supporting Information) were further measured to scrutinize the charge‐transfer kinetics at the perovskite interfaces. In contact with the perovskite, NDI‐C4F‐based sample showed much lower PL intensity and shorter average carrier lifetime (*τ_ave_*) (245.75 ns) than SnO_2_‐based ones (580.08 ns) (Figure 3d; Table S2, Supporting Information). When the perovskite was exfoliated from the ETLs, a higher PL intensity with a longer lifetime was observed in the case of NDI‐C4F in contrast to the SnO_2_ (Figure 3e; Table S3, Supporting Information), suggesting that NDI‐C4F contributed to efficient charge transport and suppressed recombination losses at the interfaces.^[^ ^38^ ^]^

In addition, the electrical properties of the perovskite films were evaluated by C‐AFM. The perovskite films on NDI‐C4F displayed much higher and uniform spatial currents across both the top surface and the buried interfaces than those of SnO_2_, indicating superior charge conductivity (Figure 3f,g). Besides, the use of NDI‐C4F enabled a lower surface roughness of the perovskite film (RMS = 15.05 nm) in comparation to SnO_2_ (RMS = 20.56) (Figure S10, Supporting Information).

### 2.3 Device Performances and Charge Carrier Dynamics

**Figure 4:**
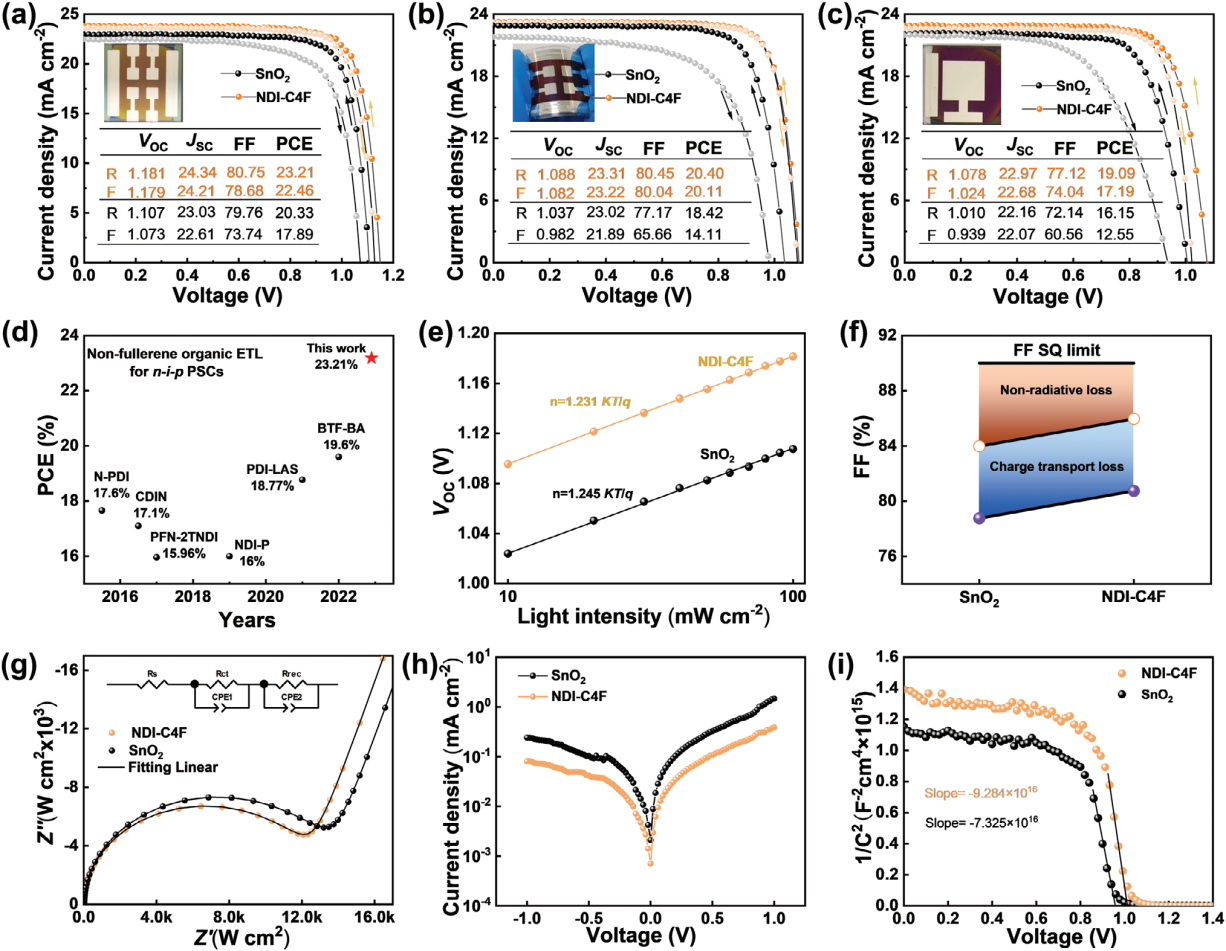
The *J–V* curves of the devices are based on a) the rigid (0.04 cm^2^), b) the flexible (0.04 cm^2^), and c) large‐area (1.004 cm^2^) PSCs with different ETLs. d) The reported efficiencies for the n‐i‐p PSCs using organic ETLs (Table S5, Supporting Information). e) Light intensity dependence of *V*_OC_ for PSCs with different ETLs. f) The Schockley‐Queisser (SQ) limit for the devices based on SnO_2_ and NDI‐C4F. g) Electrochemical impedance spectroscopy, h) dark *J–V* curves, and i) Mott–Schottky plots of the PSCs based on different ETLs.

To study the photovoltaic performance of NDI‐C4F‐based ETLs, the PSCs with a configuration of indium tin oxide (ITO)/ETL/Perovskite/Spiro‐OMeTAD/Ag were fabricated. Based on the optimization (Figure S11, Supporting Information), the best‐performing PSCs were realized when 0.5 mg mL^−1^ NDI‐C4F was applied. As presented in **Figure** **4**a, the control device with SnO_2_ delivered a moderate PCE of 20.33% with an open‐circuit voltage (*V_oc_*) of 1.107 V, a short‐circuit voltage (*J_sc_*) of 23.03 mA cm^−2^ and a fill factor (FF) of 79.76%. Encouragingly, the devices with NDI‐C4F as the ETLs achieved a higher *V_oc_* of 1.181 V, *J_sc_* of 24.34 mA cm^−2^ and FF of 80.75%, rendering a champion PCE of 23.21%, which represents the highest efficiency for n‐i‐p PSCs using organic ETLs (Figure 4d; Table S5, Supporting Information).^[^ ^19^ ^]^ As a reference, fluorine‐free NDI‐C4 as the ETLs exhibited a poor PCE of 18.21% in the devices (Figure S12, Supporting Information), which emphasizes the critical roles of fluorine groups in ETLs for performance enhancement. The devices with NDI‐C4F exhibited higher external quantum efficiencies across the entire wavelength ranges (Figure S13, Supporting Information) than that of SnO_2_, which contributed to an integrated *J_sc_* of 23.51 mA cm^−2^.

Furthermore, NDI‐C4F enabled suppressed current‐voltage hysteresis in the devices, which guarantees a reliable power output. The hysteresis index (HI) values were estimated by the following equation:^[^ ^39^ ^]^

(1) $$\mathrm{HI}=\frac{\mathrm{PC}E_{\mathrm{reverse}}-\mathrm{PC}E_{\mathrm{forward}}}{\mathrm{PC}E_{\mathrm{reverse}}}$$

Accordingly, the calculated hysteresis index was decreased from 12% in the case of SnO_2_ to 3% in the devices with NDI‐C4F (Table S4, Supporting Information), which is mostly attributed to superior interfacial contact between the perovskite and NDI‐C4F layers, the realization of improved energy level alignment and high‐quality buried interface in the perovskite layer enables effective charge transfer, consequently mitigating hysteresis phenomenon.^[^ ^40^ ^]^

To test the general applicability, we further applied NDI‐C4F in the flexible or large‐area (1.004 cm^2^) PSCs. As seen from the corresponding *J*–*V* curves (Figure 4b,c), the flexible and large‐area devices using NDI‐C4F as the ETLs achieved high PCEs of 20.40% and 19.09%, respectively, both of which were superior to those of SnO_2_‐based PSCs (18.42% for the flexible devices and 16.15% for the large‐area ones).

Notably, the enhancement of device performance for NDI‐C4F mostly benefited from the increase of *V_oc_* and FF. To gain more insights into the working mechanism of NDI‐C4F, the *V_oc_* dependence on the light intensity was measured to explore the charge dynamics in the devices (Figure 4e). The PSCs with NDI‐C4F exhibited a slope of 1.231 *KT*/*q* (K, T, and q are the Boltzmann constant, the temperature, and the elementary charge),^[^ ^41^ ^]^ which was smaller than that with SnO_2_ (1.245 *KT*/*q*), indicating NDI‐C4F favors reducing the trap‐assisted recombination. We also estimated the FF losses in contrast to the Shockley–Queisser (SQ) limit. By comparing the S‐Q FF and the calculated FF in the condition of non‐transport losses,^[^ ^42^ ^]^ the solar cells with NDI‐C4F possessed clearly lower FF losses because of reduced non‐radiative recombination (Figure 4f).

The electrical impedance spectroscopy (EIS) was measured to evaluate the charge transport and recombination at the ETL/perovskite interface (Figure 4g), and the estimated parameters are summarized in Table S6 (Supporting Information). Upon using NDI‐C4F, the charge‐transfer resistance (*R*_ct_) value was reduced to 10 796 Ω in comparison to SnO_2_ (16 154 Ω). Meanwhile, the recombination resistance (*R*_rec_) in NDI‐C4F‐based devices was estimated to be 2.16×10^5^ Ω, which was higher than that of SnO_2_ (1.49×10^5^), implying suppressed carrier recombinations.^[^ ^43^ ^]^ The dark current analysis was further examined to evaluate the charge recombination kinetics. As illustrated in Figure 4h, the device using NDI‐C4F exhibited a much lower current leakage compared to SnO_2_. Mott–Schottky analysis was implemented to investigate the charge carrier trapping and accumulating behavior at the ETL/perovskite interfaces. As plotted in Figure 4i, the build‐in potential (*V*_bi_) value for the NDI‐C4F‐based device (1.01 V) was much larger than that of SnO_2_ (0.95 V), which signifies a stronger driving force for carrier transfer and reduced interfacial carrier accumulation in the devices with NDI‐C4F.^[^ ^44^ ^]^

### 2.4 Device Stability

**Figure 5:**
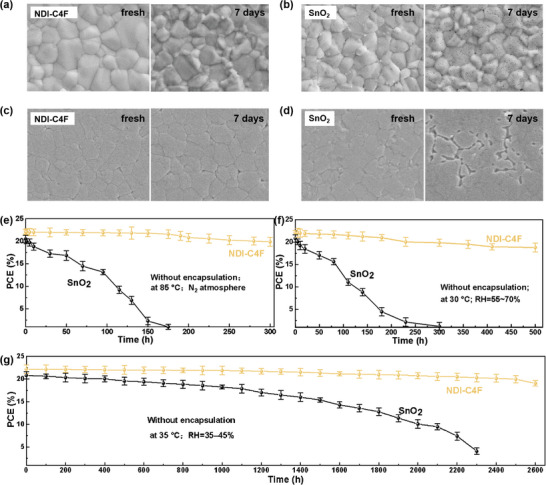
Top‐view SEM images of the perovskite films on a) NDI‐C4F and b) SnO_2_ under the illumination. SEM images of the perovskite buried interfaces with c) NDI‐C4F and d) SnO_2_ after thermal treatment. e) Thermal stability (85 ± 2 °C), f) moisture stability, and g) environmental stability of devices without encapsulation.

The influence of NDI‐C4F on the stability of perovskite films and the complete devices was systematically studied. When the perovskite films were exposed to ambient conditions (relative humidity (RH) = 35–45%) under continuous illumination (**Figure** **5**a,b), the perovskite layer on SnO_2_ showed clear degradation and large pin‐holes appeared on the film surface. Encouragingly, the perovskite film on NDI‐C4F remained dense and uniform properties for one week. Moreover, the perovskite films grown on NDI‐C4F exhibited superior thermal stability than SnO_2_, as evidenced by high‐quality buried interfaces after heating at 100 °C for one week (Figure 5c,d). We also measured the XRD and UV–vis absorption spectra of perovskite films before and after thermal treatment (Figure S14, Supporting Information), further proving the positive roles of NDI‐C4F in the aspect of enhancing film stability. Apart from the superior quality of perovskite films, the improved stabilities could be attributed to the defect passivation effects of NDI‐C4F at the buried interfaces.^[^ ^45^ ^]^

We also explored the environmental stabilities of the complete devices. In the condition of high temperature (85±2°C) and inert atmosphere, the devices with NDI‐C4F kept ≈90% of their initial PCEs after 300 h, whereas the SnO_2_‐based devices suffered from clear degradation after 50 h (Figure 5e). Under the humid conditions (RH = 65±5%), the PSCs with NDI‐C4F demonstrated impressive stability by retaining 85% of their maximal PCE after 500 h (Figure 5f). In a dry environment (RH = 35%−45%), the unencapsulated device based on NDI‐C4F maintained more than 90% of the initial efficiencies after 2600 h, in contrast, to fast decay to 40% of initial performance after 2000 h in the case of SnO_2_ (Figure 5g). All these results demonstrate the high potential of NDI‐C4F as ETLs to achieve efficient and stable PSCs.

## 3 Conclusion

The electron transport layer plays a critical role in the perovskite formation and the interfacial charge dynamics in n‐i‐p PSCs. To enhance the optoelectronic properties of perovskite buried interfaces, a representative design concept for developing fluorinated naphthalene diimide‐based organic ETMs is proposed. The designed small molecule 2,7‐bis(2,2,3,3,4,4,4‐heptafluorobutyl)benzo[*lmn*][3,8]phenanthroline‐1,3,6,8(2*H*,7*H*)‐tetraone (NDI‐C4F) was incorporated with hydrophobic fluorine units as side groups so as to form chemical coordination with the perovskite precursors. The fluorine substitution in NDI‐C4F induced retarded crystallization rate and improves the quality of perovskite buried interfaces, all of which favors the acceleration of charge separation and inhibition of carrier recombination. Consequently, the devices with NDI‐C4F yielded a remarkable efficiency of 23.21%, representing the highest reported PCE for organic ETMs in n‐i‐p devices. Moreover, the unencapsulated devices with NDI‐C4F demonstrated high reproducibility and extraordinary stability, which retained over 90% of its initial PCEs after 2600 h in air condition. Importantly, NDI‐C4F can be generally applicable to different architectures, emphasizing the reliability in device application. It reveals that fluorine‐containing side groups represent essential composition in organic ETMs for the optimization of perovskite crystallization kinetics and the buried interfaces. This study would provide a new avenue for the design of efficient and stable organic ETMs for n‐i‐p PSCs.

## Conflict of Interest

The authors declare no conflict of interest.

## Supporting information

Supporting Information

## Data Availability

The data that support the findings of this study are available from the corresponding author upon reasonable request.

## References

[1] R. He , W. Wang , Z. Yi , F. Lang , C. Chen , J. Luo , J. Zhu , J. Thiesbrummel , S. Shah , K. Wei , Y. Luo , C. Wang , H. Lai , H. Huang , J. Zhou , B. Zou , X. Yin , S. Ren , X. Hao , L. Wu , J. Zhang , J. Zhang , M. Stolterfoht , F. Fu , W. Tang , D. Zhao , Nature 2023, 618, 80. 36990110 10.1038/s41586-023-05992-y

[2] M. Jeong , I. W. Choi , K. Yim , S. Jeong , M. Kim , S. J. Choi , Y. Cho , J. H. An , H. B. Kim , Y. Jo , S. H. Kang , J. H. Bae , C. W. Lee , D. S. Kim , C. Yang , Nat. Photonics 2022, 16, 119.

[3] J. Park , J. Kim , H. S. Yun , M. J. Paik , E. Noh , H. J. Mun , M. G. Kim , T. J. Shin , S. I. Seok , Nature 2023, 616, 724. 36796426 10.1038/s41586-023-05825-y

[4] X. Zheng , Z. Li , Y. Zhang , M. Chen , T. Liu , C. Xiao , D. Gao , J. B. Patel , D. Kuciauskas , A. Magomedov , R. A. Scheidt , X. Wang , S. P. Harvey , Z. Dai , C. Zhang , D. Morales , H. Pruett , B. M. Wieliczka , A. R. Kirmani , N. P. Padture , K. R. Graham , Y. Yan , M. K. Nazeeruddin , M. D. McGehee , Z. Zhu , J. M. Luther , Nat. Energy 2023, 8, 462.

[5] National Renewable Energy Laboratory, Best research‐cell efficiency chart; https://www.nrel.gov/pv/cell‐efficiency.html, (accessed: June 2024).

[6] X. Huang , F. Cao , S. Zhan , Q. Feng , M. Zhu , Z. Su , X. Gao , J. Yin , J. Li , N. Zheng , B. Wu , Joule 2023, 7, 1556.

[7] S. You , H. Zeng , Y. Liu , B. Han , M. Li H , L. Li , X. Zheng , R. Guo H , L. Luo , Z. Li , C. Zhang , R. Liu , Y. Zhao , S. Zhang , Q. Peng , T. Wang , Q. Chen , F. T. Eickemeter , B. Carlsen , S. M. Zakeeruddin , L. Mai , Y. Rong , M. Gratzel , X. Li , Science 2023, 379, 288. 36656941 10.1126/science.add8786

[8] C. Luo , G. Zheng , F. Gao , X. Wang , C. Zhan , X. Gao , Q. Zhao , Nat. Photonics 2023, 17, 856.

[9] Z. Lin , W. Zhang , Q. Cai , X. Xu , H. Dong , C. Mu , J. P. Zhang , Adv. Sci. (Weinh) 2021, 8, e2102845. 34633769 10.1002/advs.202102845 PMC8596138

[10] H. Pan , X. Zhao , X. Gong , H. Li , N. H. Ladi , X. L. Zhang , W. Huang , S. Ahmad , L. Ding , Y. Shen , M. Wang , Y. Fu , Mater. Horiz. 2020, 7, 2276.

[11] G. Bahuguna , M. Verma , R. Gupta , J. Mater. Chem. A 2021, 9, 19965.

[12] J. Gong , Y. Cui , F. Li , M. Liu , Small Sci. 2023, 3, 2200108. 10.1002/smsc.202200108 PMC11935800 40212913

[13] J. Chen , J. Zhang , C. Huang , Z. Bi , X. Xu , H. Yu , Chem. Eng. J. 2021, 410, 128436.

[14] J. Wang , K. Datta , C. H. L. Weijtens , M. M. Wienk , R. A. J. Janssen , Adv. Funct. Mater. 2019, 29, 1905883.

[15] W. Wang , J. Zhou , W. Tang , J. Mater. Chem. A 2022, 10, 1150.

[16] M. A. Jameel , T. C.‐J. Yang , G. J. Wilson , R. A. Evans , A. Gupta , S. J. Langford , J. Mater. Chem. A 2021, 9, 27170.

[17] Q. Zhou , Y. Gao , C. Cai , Z. Zhang , J. Xu , Z. Yuan , P. Gao , Angew Chem Int Ed Engl 2021, 60, 8303. 33492689 10.1002/anie.202017148

[18] M. Liu , Y. Jiang , D. Liu , J. Wang , Z. Ren , T. P. Russell , Y. Liu , ACS Energy Lett. 2021, 6, 3228.

[19] N. Fan , Y. Wang , C. Zhang , G. Zhu , G. Du , K. Wei , J. Deng , Z. Luo , L. Yang , J. Zhang , J. Mater. Chem. A 2022, 10, 8911.

[20] M. Liu , P. Fan , Q. Hu , T. P. Russell , Y. Liu , Angew Chem Int Ed Engl 2020, 59, 18131. 32558039 10.1002/anie.202004432

[21] B. Chen , P. N. Rudd , S. Yang , Y. Yuan , J. Huang , Chem. Soc. Rev. 2019, 48, 3842. 31187791 10.1039/c8cs00853a

[22] J. Yang , C. Liu , C. Cai , X. Hu , Z. Huang , X. Duan , X. Meng , Z. Yuan , L. Tan , Y. Chen , Adv. Energy Mater. 2019, 9, 1900198.

[23] N. A. N. Ouedraogo , H. Yan , C. B. Han , Y. Zhang , Small 2021, 17, e2004081. 33522104 10.1002/smll.202004081

[24] J. Zhou , X. Yin , Z. Dong , A. Ali , Z. Song , N. Shrestha , S. S. Bista , Q. Bao , R. J. Ellingson , Y. Yan , W. Tang , Angew Chem Int Ed Engl 2019, 58, 13717. 31286608 10.1002/anie.201905624

[25] T. Wu , Y. Wang , X. Li , Y. Wu , X. Meng , D. Cui , X. Yang , L. Han , Adv. Energy Mater. 2019, 9, 1803766.

[26] Z. Guo , A. K. Jena , G. M. Kim , T. Miyasaka , Energy Environ. Sci. 2022, 15, 3171.

[27] W. Wang , Z. Lin , S. Gao , W. Zhu , X. Song , W. Tang , Adv. Functional Mater. 2023, 30, 2003619.

[28] Y. Li , H. Wu , W. Qi , X. Zhou , J. Li , J. Cheng , Y. Zhao , Y. Li , X. Zhang , Nano Energy 2020, 77, 105237.

[29] D. Wei , F. Ma , R. Wang , S. Dou , P. Cui , H. Huang , J. Ji , E. Jia , X. Jia , S. Sajid , A. M. Elseman , L. Chu , Y. Li , B. Jiang , J. Qiao , Y. Yuan , M. Li , Adv. Mater. 2018, 30, 1707583. 10.1002/adma.201707583 29938843

[30] E. Bi , Z. Song , C. Li , Z. Wu , Y. Yan , Trends Chem 2021, 3, 575.

[31] A. Kulkarni , R. Sarkar , S. Akel , M. Häser , B. Klingebiel , M. Wuttig , S. Wiegand , S. Chakraborty , M. Saliba , T. Kirchartz , Adv. Functional Mater. 2023, 33, 2305812.

[32] Y. Zhong , J. Yang , X. Wang , Y. Liu , Q. Cai , L. Tan , Y. Chen , Adv. Mater. 2023, 35, 2302552. 10.1002/adma.202302552 37067957

[33] W. Chen , X. Li , Y. Li , Y. Li , Energy Environ. Sci. 2020, 13, 1971.

[34] C. Wu , W. Fang , Q. Cheng , J. Wan , R. Wen , Y. Wang , Y. Song , M. Li , Angew Chem Int Ed Engl 2022, 61, e202210970. 36050600 10.1002/anie.202210970

[35] K. Wei , L. Yang , J. Deng , Z. Luo , X. Zhang , J. Zhang , ACS Appl. Energy Mater. 2022, 5, 7458.

[36] L. Li , M. Wei , V. Carnevali , H. Zeng , M. Zeng , R. Liu , N. Lempesis , F. T. Eickemeyer , L. Luo , L. Agosta , M. Dankl , S. M. Zakeeruddin , U. Roethlisberger , M. Gratzel , Y. Rong , X. Li , Adv. Mater. 2023, 36, 2303869. 10.1002/adma.202303869 37632843

[37] A. Ullah , K. H. Park , H. D. Nguyen , Y. Siddique , S. F. A. Shah , H. Tran , S. Park , S. I. Lee , K. K. Lee , C. H. Han , K. Kim , S. Ahn , I. Jeong , Y. S. Park , S. Hong , Adv. Energy Mater. 2021, 12, 2103175.

[38] J. Deng , K. Wei , L. Yang , L. Lin , Y. Xiao , X. Cai , C. Zhang , D. Wu , X. Zhang , J. Zhang , Adv. Mater. 2023, 35, 2300233. 10.1002/adma.202300233 37021877

[39] T. Duong , Y. Wu , H. Shen , J. Peng , X. Fu , D. Jacobs , E. C. Wang , T. C. Kho , K. C. Fong , M. Stocks , E. Franklin , A. Blakers , N. Zin , K. McIntosh , W. Li , Y. B. Cheng , T. P. White , K. Weber , K. Catchpole , Adv. Energy Mater. 2017, 7, 1700228.

[40] D. H. Kang , N. G. Park , Adv. Mater. 2019, 31, 1805214.

[41] W. Wang , K. Wei , L. Yang , J. Deng , J. Zhang , W. Tang , Mater. Horiz. 2023, 10, 2609. 37097145 10.1039/d3mh00219e

[42] J. Wang , J. Zhang , Y. Zhou , H. Liu , Q. Xue , X. Li , C. Chueh , H. Yip , Z. Zhu , A. Jen , Nat. Commun. 2020, 11, 177. 31924773 10.1038/s41467-019-13909-5 PMC6954256

[43] H. Huang , P. Cui , Y. Chen , L. Yan , X. Yue , S. Qu , X. Wang , S. Du , B. Liu , Q. Zhang , Z. Lan , Y. Yang , J. Ji , X. Zhao , Y. Li , X. Wang , X. Ding , M. Li , Joule 2022, 6, 2186.

[44] W. Wang , X. Liu , J. Wang , C. Chen , J. Yu , D. Zhao , W. Tang , Adv. Energy Mater. 2023, 13, 2300694.

[45] J. Zhang , B. Yu , Y. Sun , H. Yu , Adv. Energy Mater. 2023, 13, 2300382.

