# Supplementary material for: Fluorinated Naphthalene Diimides as Buried Electron Transport Materials Achieve Over 23% Efficient Perovskite Solar Cells
Source: Adv Sci (Weinh). 2024 Jul 23;11(36):2403735. doi: 10.1002/advs.202403735 (PMC11423218; doi:10.1002/advs.202403735)
Supplement: Supplementary file 1 — Supporting Information [file ADVS-11-2403735-s001.docx]

Supplementary Information

**Supplementary note 1**

1. Synthesis details of ETLs

All reagents and solvents were purchased from J&K, energy chemical or Sigma-Aldrich and they were used as bought without further purification, unless otherwise stated. Structures of the synthesized compounds were confirmed by means of ^1^H, ^13^C nuclear magnetic resonance spectroscopy (NMR) and matrix-assisted laser desorption/ionization time-of-flight (MALDI-TOF) mass spectra. The synthetic route is depicted in Scheme S1, and the detailed synthetic procedures are listed as below.


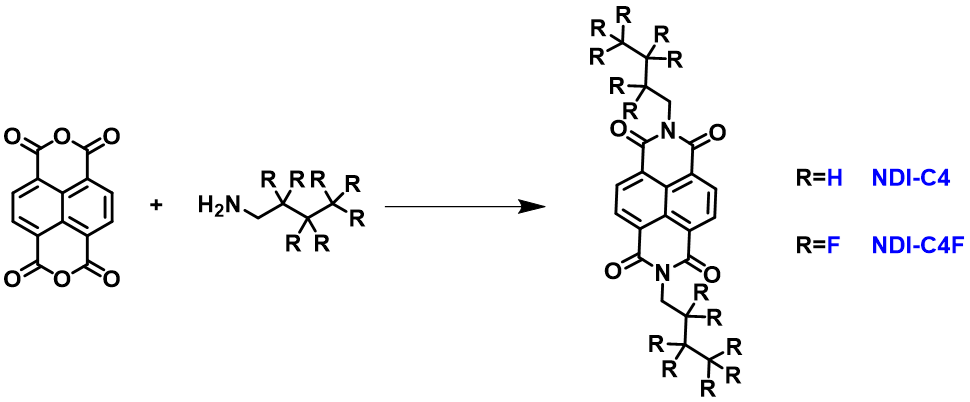


**Scheme S1**. Synthetic routes of **NDI-C4** and **NDI-C4F**.

Synthesis of 2,7-dibutylbenzo[*lmn*][3,8]phenanthroline-1,3,6,8(2*H*,7*H*)-tetraone (NDI-C4)

1,4,5,8-naphthalenetetracarboxylic dianhydride (2.00 g, 7.46 mmol) was dissolved in 20 mL *N*, *N*-dimethylformamide (DMF) and stirred for 30 min at 90 °C. Then the solution of *n*-butylamine (1.20 g, 16.41 mmol) in 10 mL DMF was added dropwise. The reaction mixture was heated at reflux for 24 h. After completion of the reaction, extraction was done with dichloromethane. The organic layer was dried over by using anhydrous MgSO_4_ and the solvent was distilled off under reduced pressure. The crude product was purified by column chromatography (silica gel, petroleum ether/dichloromethane, v/v, 1/2) to afford NDI-C4 as a light pink solid (1.68 g, 60%). ^1^H NMR (500 MHz, CDCl_3_) : 8.75 (s, 4H), 4.22-4.17 (m, 4H), 1.76-1.69 (m, 4H), 1.45 (m, *J* = 14.9, 7.5 Hz, 4H), 0.99 (t, *J* = 7.4 Hz, 6H). ^13^C NMR (125 MHz, CDCl_3_): 162.96, 131.06, 126.79, 126.76, 40.91, 30.32, 20.51, 13.98. MALDI-TOF (m/z): [M]^+^ calculated for C_22_H_22_N_2_O_4_: 378.1580, found: 378.1613.

**Synthesis of 2,7-bis(2,2,3,3,4,4,4-heptafluorobutyl)benzo[*lmn*][3,8]phenanthroline-1,3,6,8(2*H*,7*H*)-tetraone (NDI-C4F)**

1,4,5,8-naphthalenetetracarboxylic dianhydride (0.7 g, 2.61 mmol) was dissolved in 15 mL *N*, *N*-dimethylformamide (DMF) and stirred for 30 min at 90 °C. Then the solution of 2,2,3,3,4,4,4-heptafluorobutylamine (1.09 g, 5.48 mmol) in 10 mL DMF was added dropwise. The reaction mixture was heated at reflux for 24 h. After completion of the reaction, extraction was done with dichloromethane. The organic layer was dried over by using anhydrous MgSO_4_ and the solvent was distilled off under reduced pressure. The crude product was purified by column chromatography (silica gel, petroleum ether/dichloromethane, v/v, 1/3) to afford NDI-C4F as a light pink solid (0.87 g, 53%). ^1^H NMR (500 MHz, CDCl_3_): 8.86 (s, 4H), 5.03 (t, *J* = 15.3 Hz, 4H). ^13^C NMR (125 MHz, CDCl_3_): 162.45, 132.07, 127.21, 126.56, 115.00, 112.93, 39.01, 38.83, 38.65. MALDI-TOF (m/z): [M]^+^ calculated for C_22_H_8_F_14_N_2_O_4_: 630.0261, found: 630.0287.


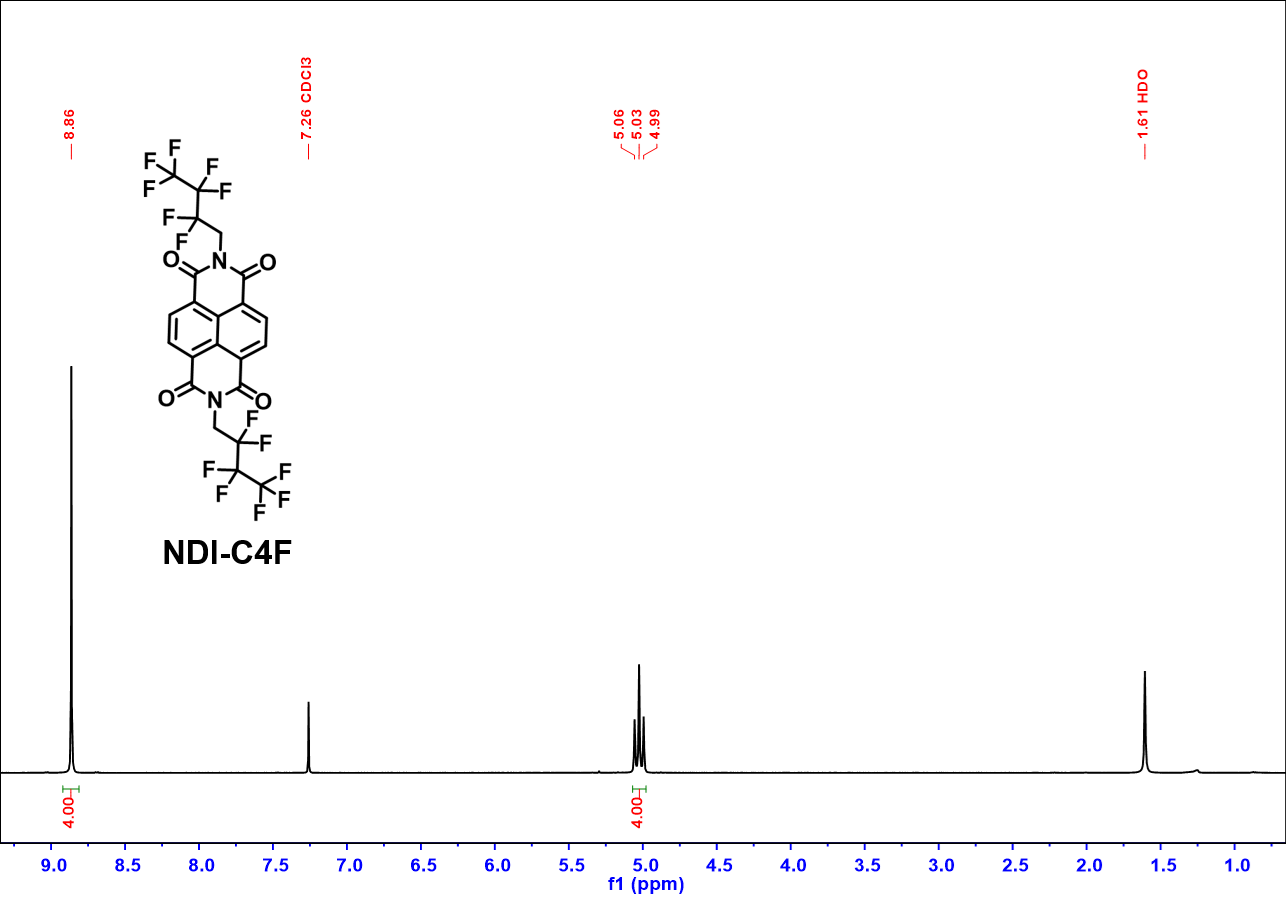
 Figure S1. ^1^H NMR spectrum of NDI-C4F.


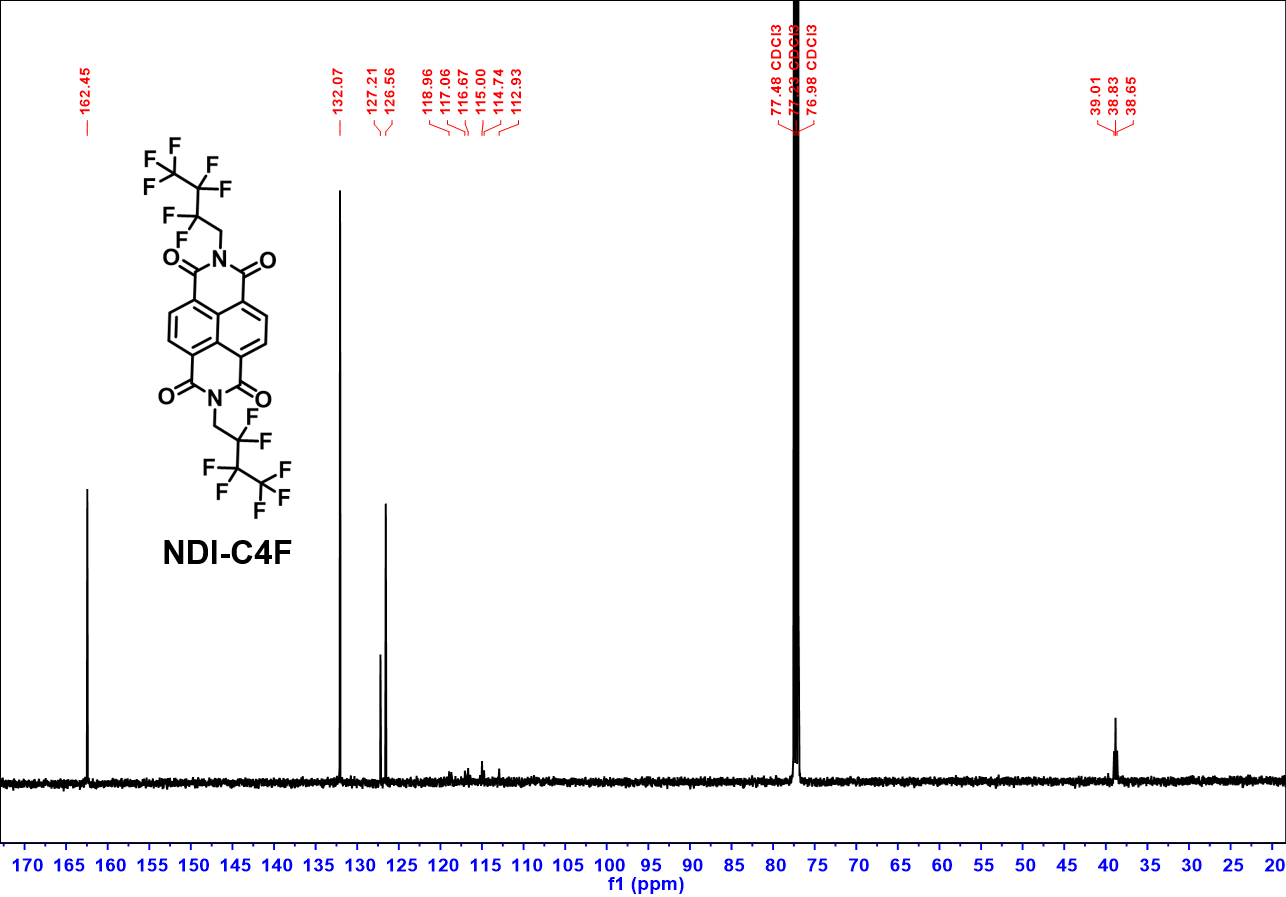
 Figure S2. ^13^C NMR spectrum of NDI-C4F.


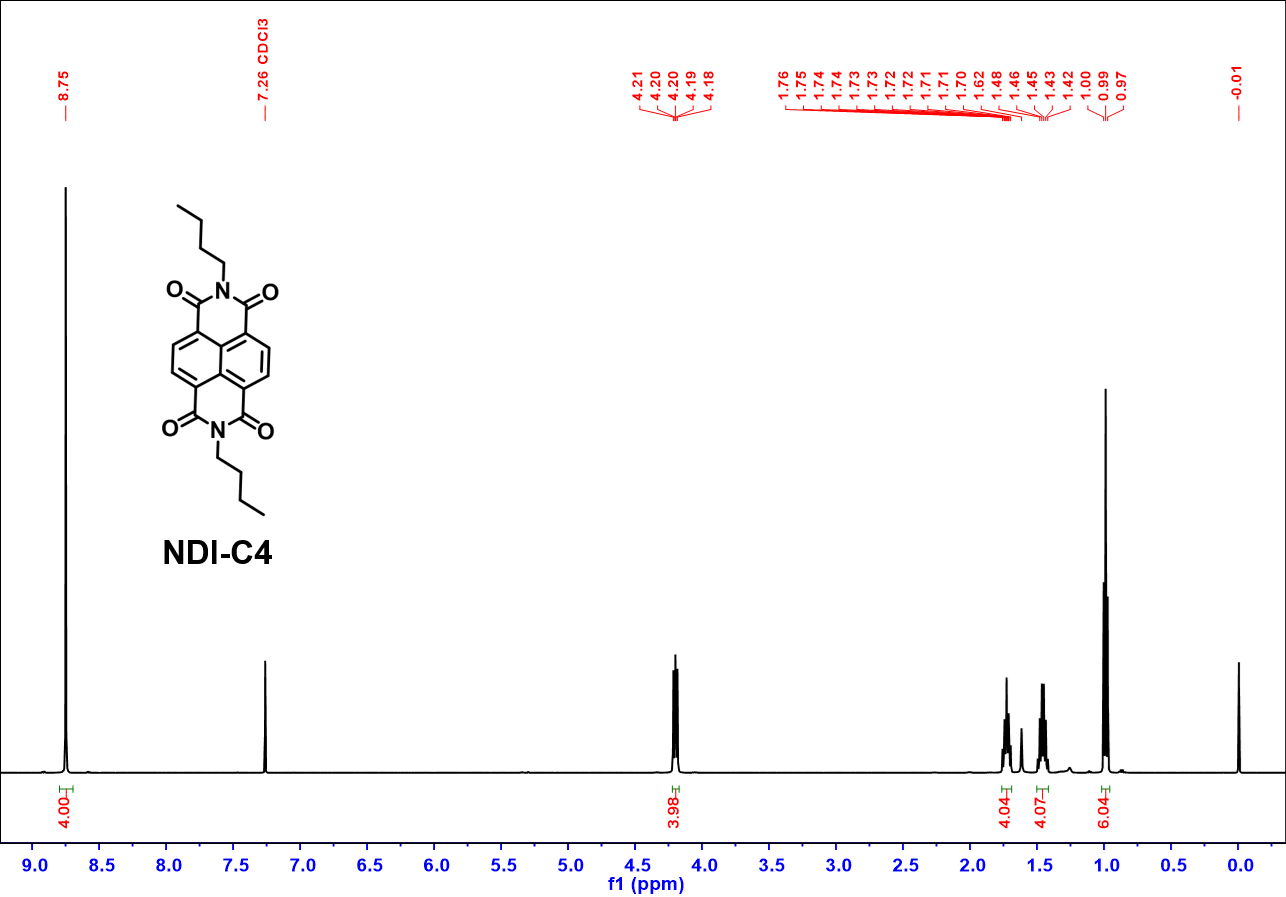
 Figure S3. ^1^H NMR spectrum of NDI-C4.


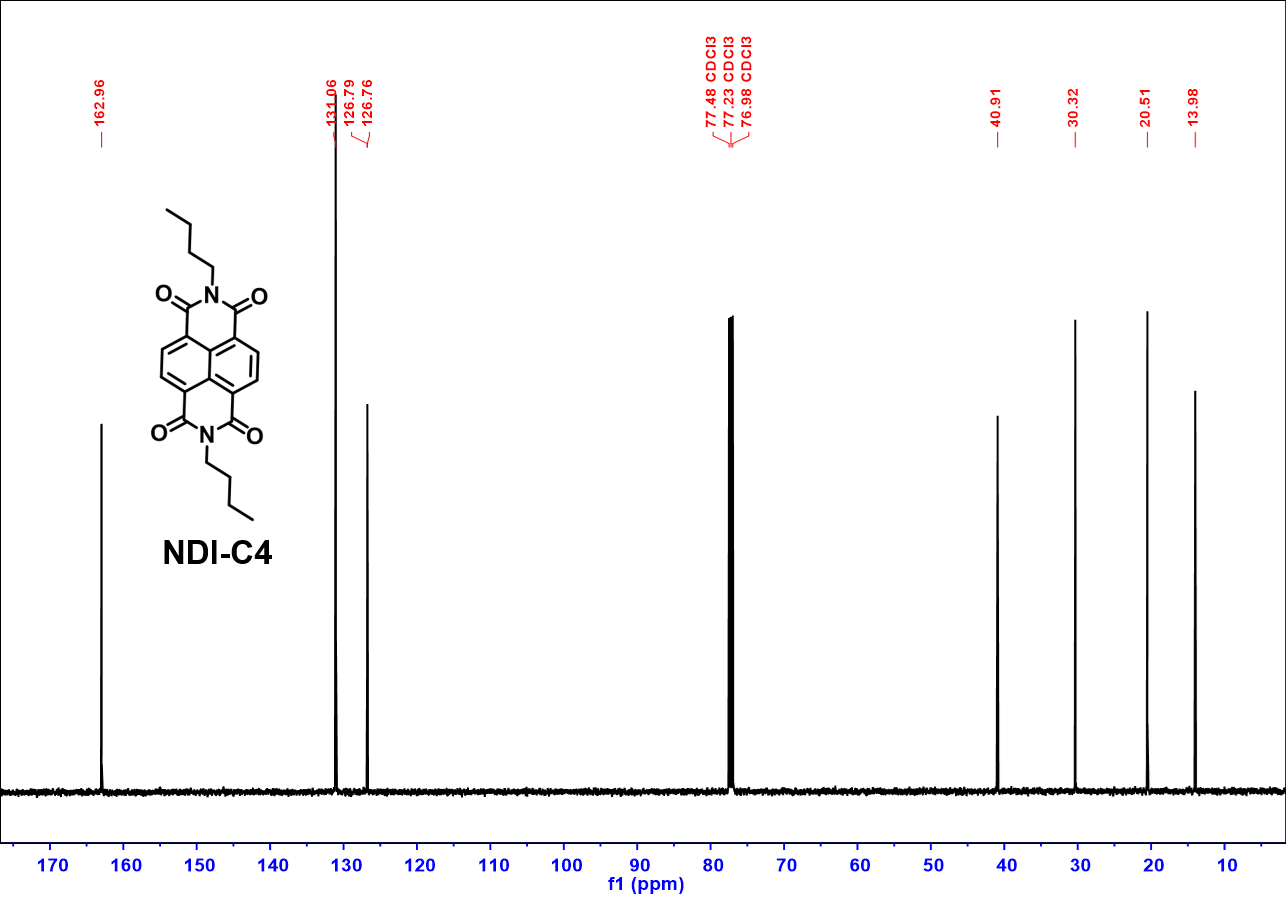


Figure S4. ^13^C NMR spectrum of NDI-C4.

1. Experimental details

1.1. Materials

All reagents and solvents were purchased commercially and used as received without further purification, unless otherwise stated. Methylammonium iodide (MAI), Cesium iodide (CsI), methylammonium chloride (MACl), formamidinium bromide (FABr), 2, 2’, 7, 7’-tetrakis(*N, N*-di-p-methoxy-phenylamine)-9, 9’-spirobifuorene (Spiro-OMeTAD), Indium tin oxide film (ITO) were purchased from Advanced Election Technology Co., LtD). Dimethyl sulfoxide (DMSO), *N, N*-dimethylformamide (DMF), 4-tert-butyl pyridine (*t*BP), chlorobenzene (CB), Ethyl acetate (EA) were purchased from Sigma-Aldrich. Lead (II) iodide (PbI_2_) and Lead (II) bromide (PbBr_2_) were purchased from TCI. Lithium bis(trifluoromethanesulfonyl)imide (LiTFSI) and iso-butylammonium bromide (*i*-BABr) were purchased from Xi'an Paulette. Acetonitrile (ACN) and isopropanol (IPA) were purchased from Energy Chemical. Formamidinium iodide (FAI) was synthesized in our own laboratory. Tin(IV) oxide (SnO_2_, 15% in H_2_O colloidal dispersion) was purchased from Xi'an Yuri Solar Co., Ltd.

1.2. Fabrication of PSCs

The PSCs had an *n–i–p* structure: glass/ITO/ETL/perovskite/*i*-BABr/Spiro-OMeTAD HTL/Ag contact.

The ITO glass substrates were cleaned with glass cleaning fluid, deionized water, acetone and isopropyl alcohol for 15 min, respectively and then dried by compressed air. The clean ITO glass was treated with UV-Ozone for 15 min to promote wettability. The SnO_2_ (15 wt%) nanoparticle solution was mixed with deionized water in a volume ratio of 1:3 and stirred for 5 min. To prepare SnO_2_ film, the solution was spin-coated onto the ITO substrates at 2500 rpm for 30 s and annealed at 200 °C for 40 min to prepare electron transport layer. To prepare NDI-C4F films, the NDI derivatives were mixed with chlorobenzene at different concentrations: 0, 0.25, 0.5, 0.75, 1 mg/mL. The optimal concentration ratio of NDI-C4F/CB was 0.5 mg/mL. The ETL were fabricated on top of ITO substrates by spin coating at 3000 rpm for 30 s. After cooling to room temperature, the substrates were treated with UV-ozone again (only for SnO_2_ films). To fabricate MA_0.03_Cs_0.07_FA_0.9_Pb (I_0.92_Br_0.08_)_3_ perovskite solution, 12.8 mg of MAI, 27.1 mg of FABr, 49.2 mg of CsI, 63.8 mg of MACl, 79.2 mg of PbBr_2_, 380.8 mg of FAI and 1182.4mg of PbI_2_ were mixed in a DMSO/DMF mixture (4:1) and then stirred in the glove box overnight. The perovskite solution was filtered through a 0.22 µm polytetrafluoroethylene (PTFE) filter before use. Perovskite films were deposited in the glovebox with ethyl acetate as the antisolvent. In detail, the well-filtered 40 μL perovskite precursor solution was dropped onto the ETL substrates and spin-coated at 1000 rpm. for 10 s and 4000 rpm. for 30 s. We quickly dropped 260 µl of ethyl acetate onto the substrates 20 s before the end of the program. The samples were immediately placed on a preheated hot plate and annealed at 100 °C for 40 mins. The *i*-BABr solution (2.3 mg/mL in IPA) was dynamically spin coated on the perovskite film and annealed at 100 °C for 5 minutes as described in the literature^[1]^.The Spiro-OMeTAD precursor solution was prepared by dissolving 72.5 mg Spiro-OMeTAD, 28.8 µL *t*BP and 17.5 µL LiTFSI solution (520 mg/mL in acetonitrile) in 1 mL chlorobenzene. The Spiro-OMeTAD film was deposited on top of the perovskite film by spin coating at 3000 r.p.m. for 30 s. The samples were then placed in a humidity-controlled cabinet filled with air (RH, <18%, 30 °C) for 24 h to ensure sufficient oxidation of the doped Spiro-OMeTAD hole-transport layer before evaporation of the Ag contact. Finally, A 80nm layer of Ag film was thermally evaporated as the contact electrode using a 0.09 cm^2^ shadow mask (PD-400S, Wuhan PDVacuum Technologies Co., Ltd).

**1.3. Characterizations**

The ^1^H NMR and ^13^C NMR spectra were recorded on a Bruker AVANCE 500 MHz spectrometer with deuterated chloroform (CDCl_3_) as the solvent and teramethylsilane (TMS) as the internal reference. Mass spectra were collected using GCT-MS EI and Bruker Daltonics Biflex III MALDI-TOF Analyzer in the MALDI mode. The software of VMD was applied to map molecular surface electrostatic potential. The electron density level of Van der Waals surface was defined to be 0.001 e Bohr^-3^, and all molecular size information was calculated by Multiwfn.

The space charge limiting current (SCLC), capacitance-voltage (*C-V*), linear scanning voltage (LSV), Mott-Schottky analysis and electrochemical impedance (EIS) were performed in the dark by electrochemical workstation (CHI600E). The current density–voltage (*J–V*) measurements were performed in N_2_ atmosphere under an illumination power of 100 mW cm^−2^. The devices were measured both in reverse scan (1.2 V to −0.1 V) and forward scan (−0.1 V to 1.2 V) with a scan rate of 0.1 V s^−1^ with an effective active area of 0.04 cm^2^ (rigid and flexible device) and 1.004 cm^2^ (large area device). The long-term operational stability test was measured at the maximum power point (MPP) for the unencapsulated devices (Ezhou Zhong Neng Optoelectronics Co., Ltd.). The incident photon-to-current conversion efficiency (IPCE) measurement was obtained on an IPCE measurement system (Model, QE-R, made in Taiwan, China). For exfoliation process of the perovskite films: PMMA (Sigma-Aldrich) precursor was prepared by dissolving 0.4g PMMA in 1mL CB. Epoxy precursor was prepared by mixing diglycidyl ether bisphenol A type (Sigma-Aldrich), n-octylamine (Sigma-Aldrich) and m-xylylenediamine (Sigma-Aldrich) with a molar ratio of 4:2:1. PMMA and epoxy layer were blade coated on the perpated perovskite film in a sequential order. In order to accelerate the cross-link process of epoxy, the coated substrate was annealed at 70 °C for 10 min. After 12 hours, the epoxy was completely solidified at room temperture. Finally, perovskite film was exfoliated from glass/ITO substrate by a glass nipping plier. Capacitance–voltage measurement was conducted to study the interfacial electronic structures in devices:

$$\left( \frac{C}{A} \right)^{2}=\frac{q\varepsilon\varepsilon_{0}N}{2(V_{bi}-V)}$$

where *V* is the applied bias, *A* is the active area, e is the charge of the element, *ɛ* is the static permittivity of the perovskite, *ɛ_0_* is the vacuum permittivity, and *N* is the carrier concentration. The X-axis intersection represents the built-in potential *V_bi_*, and the slope reflects the carrier density of the interface^[2]^. (The PSCs with an *n–i–p* structure: glass/ITO/ETL/perovskite/*i*-BABr/Spiro-OMeTAD/Ag contact.)

Top-view optical images were taken by the Fluorescence polarizing microscope (Leica DM6000B). Secondary electron images (SEMs) were recorded using a field-emission scanning electron microscope (SU-70). The X-ray diffraction (XRD) pattern was measured by Bruker-axs XRD with a Cu Kα radiation source. The scanning kelvin probe microscopy (SKPM) and conductive atomic force microscopy (C-AFM) were measured by Cypher ES (Oxford Instruments Asylum Research) via AC mode at 2.44 Hz with ASYELEC.01-R2 probe. Photoluminescence (PL) and time resolved photoluminescence (TRPL) spectra were obtained by using FLS1000 (Edinburgh instruments) with an excitation at 460 and 450 nm, respectively.

The ultraviolet-visible (UV-vis) absorption spectra were collected on a UV-visible spectrophotometer (Shimadzu UV-2550). (To prepare SnO_2_ film, the solution was spin-coated onto the ITO substrates at 2500 rpm for 30 s and annealed at 200 °C for 40 min. To prepare NDI-C4F film, the solution (0.5 mg/mL in chlorobenzene) was spin coated on top of ITO substrates at 3000 rpm for 30 s.)

Ultraviolet photoelectron spectroscopy (UPS) was performed by PHI 5000 VersaProbe III with He I source (21.22 eV) under an applied negative bias of 5.0 V. The water contact angle was obtained by a video optical contact angle measuring instrument (DSA-20) with a drop of ultrapure water (1 µL). (For UPS and water contace angle measurement, the characterization ETL films were fabricated by spin coating 2500 rpm for 30 s and annealed at 200 °C for 40 min (SnO_2_ based ETL), 3000 rpm for 30 s for NDI-C4F and NDI-C4 based film ( 0.5 mg/mL in chlorobenzene).

XPS spectra were measured using a Thermo-Fisher ESCA-LAB 250Xi system with a monochromatized Al Ka (for XPS mode) under the pressure of 5.0x10^-7^ Pa. (For XPS measurement, the characterization NDI-C4F film was prepared by spin coating on top of ITO substrates at 3000 rpm for 30 s (0.5mg/mL in chlorobenzene).

The DFT calculations in this research are performed using the Vienna ab initio simulation software (VASP) ^[3]^. To simulate electron exchange-related interactions, the Perdew–Burke–Ernzerhof (PBE) ^[3]^ functional is utilized, and the projection enhanced wave (PAW) approach is used for electron–ion–nucleus interactions. In order to handle van der Waals interactions in perovskites, we employ the Grimme DFT-D3 approach with Becke-Johnson damping. Geometry optimization is carried out with the Γ-centered 2×2×1 Monkhorst−Pack k-point mesh and the 400 eV plane wave energy cutoff. The geometric structure is regarded as convergent when the energy difference between all ions is smaller than -10^-4^ eV.


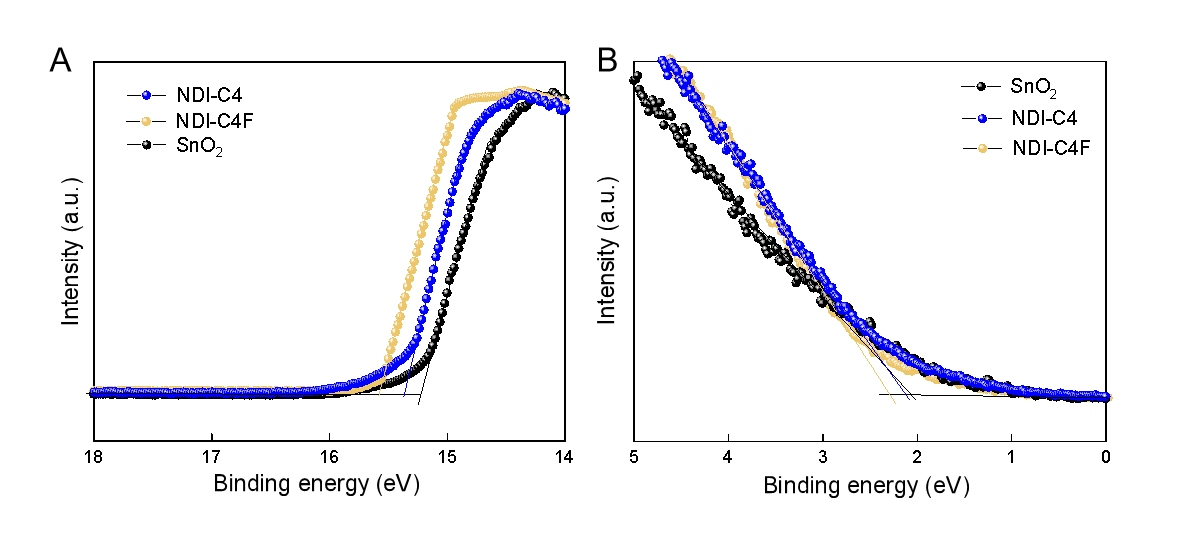


**Fig. S5 | UPS results of different ETLs deposited on ITO.** Secondary electron cut-off (A), and the energy band edge of (B) SnO_2_, NDI-C4 and NDI-C4F based ETLs. (the excitation energy is 21.22eV).


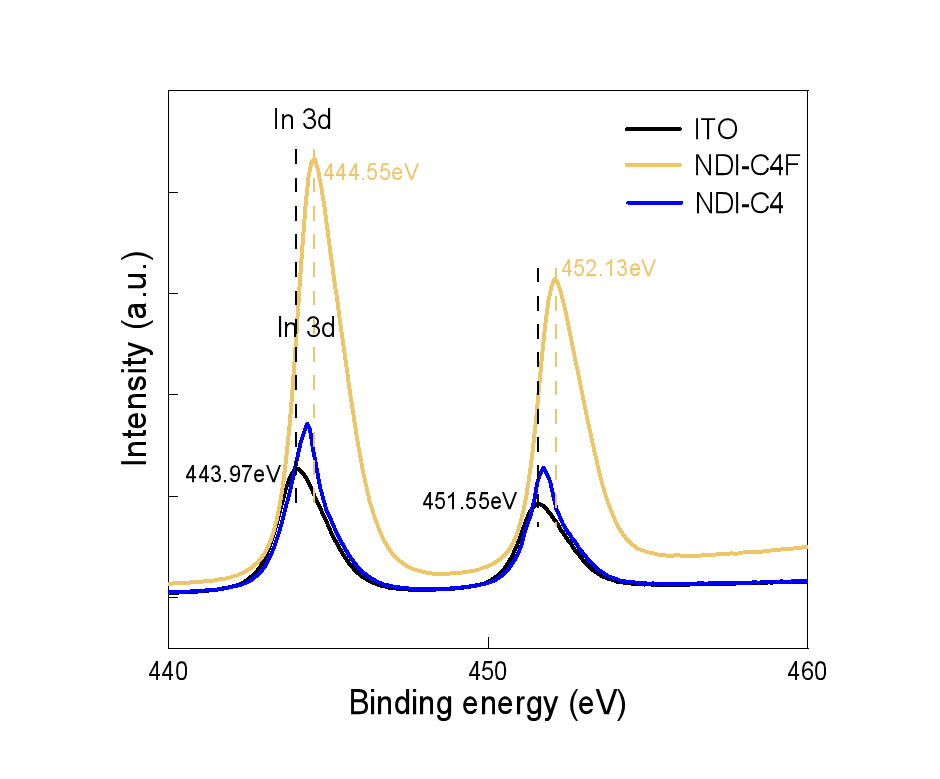


**Fig. S6 |** In 3d XPS spectra of the different ETLs deposited on the ITO substrate.


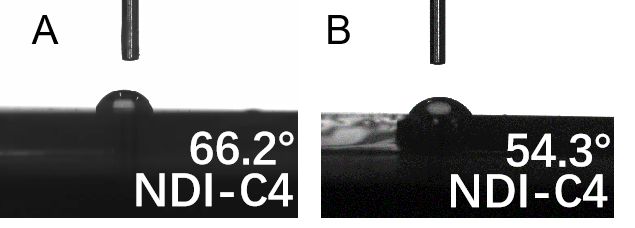


**Fig. S7 |** **Perovskite (A) and water (B) contact angle with the NDI-C4 films**.


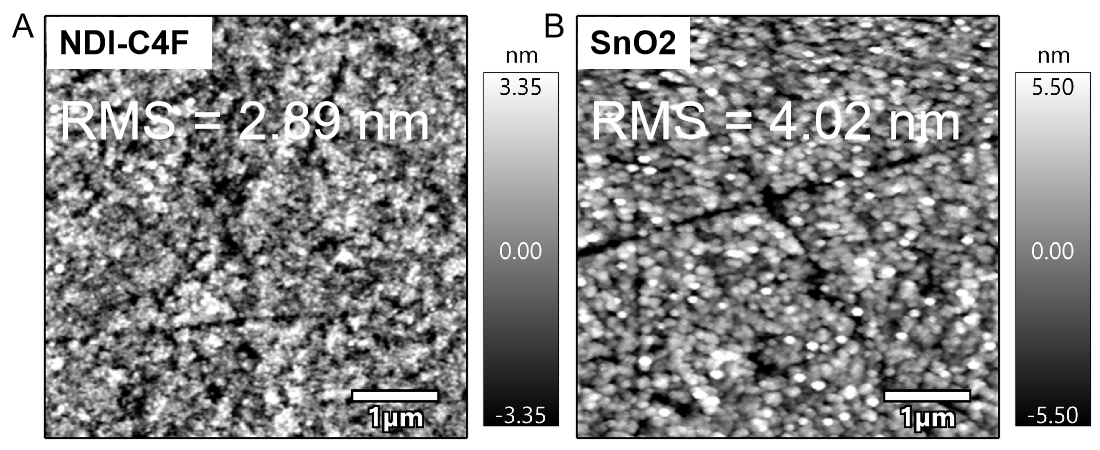


**Fig. S8 | Corresponding AFM results of different ETLs deposited on ITO substrates.** AFM images of NDI-C4F (A) and SnO_2_ (B) ETLs.


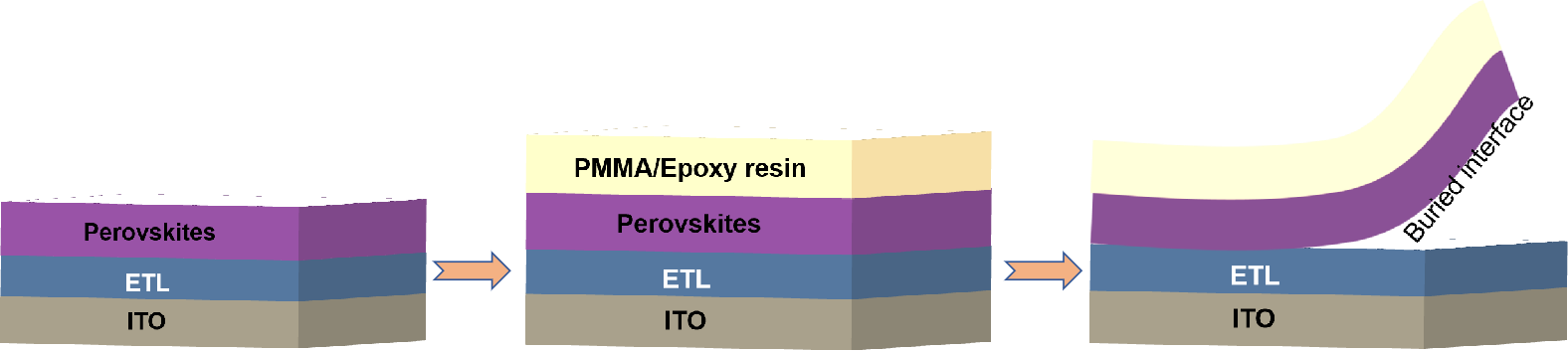


**Fig. S9 |**  Schematic diagram of exfoliation of perovskite film from bottom electrode.


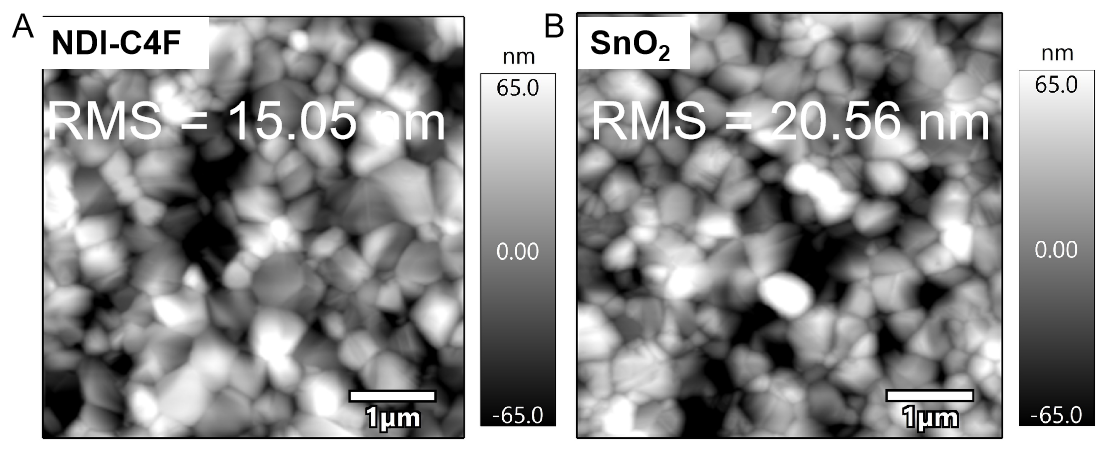


**Fig. S10 |** The top-view AFM images of the perovskite films deposited on NDI-C4F and SnO_2_.


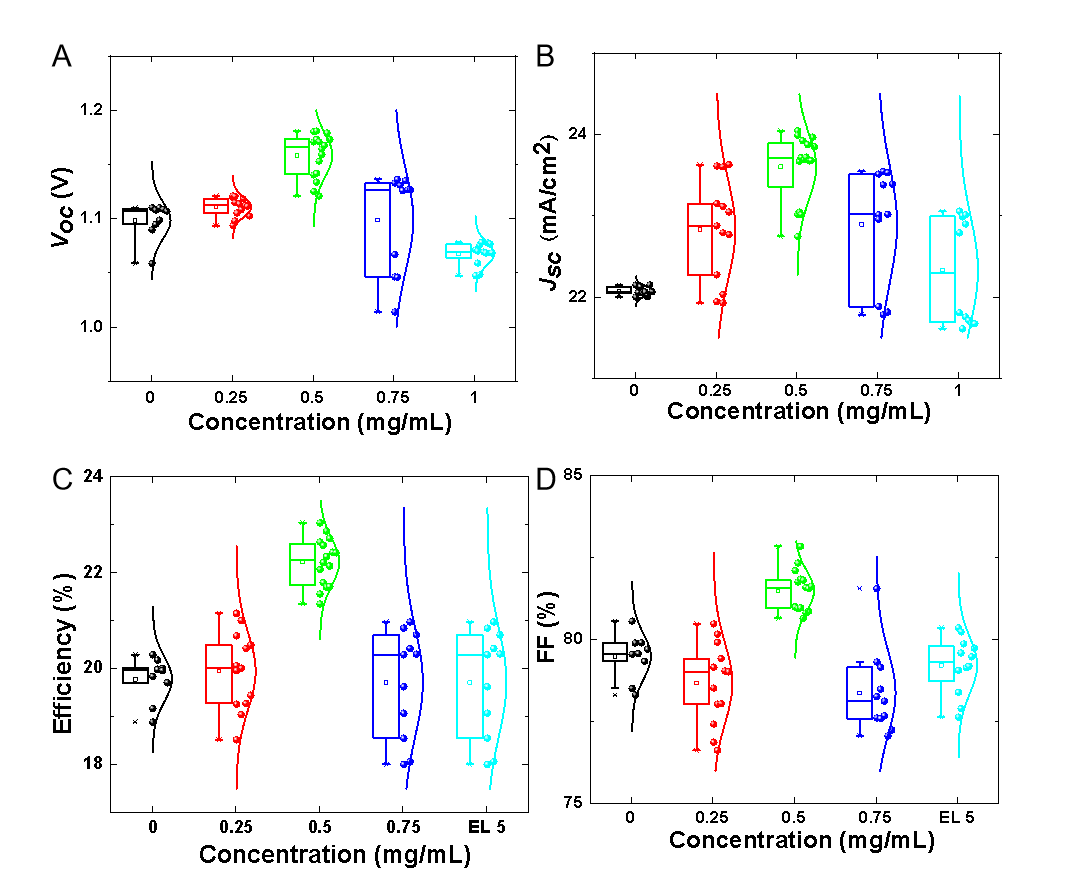


**Fig. S11 |** Statistical distribution diagram of the PCE, *V_oc_*, FF and *J_sc_* of rigid PSCs prepared on the ETLs with different concentration of NDI-C4F. (the NDI-C4F was mixed with chlorobenzene at different concentrations: 0, 0.25, 0.5, 0.75, 1 mg/mL.)


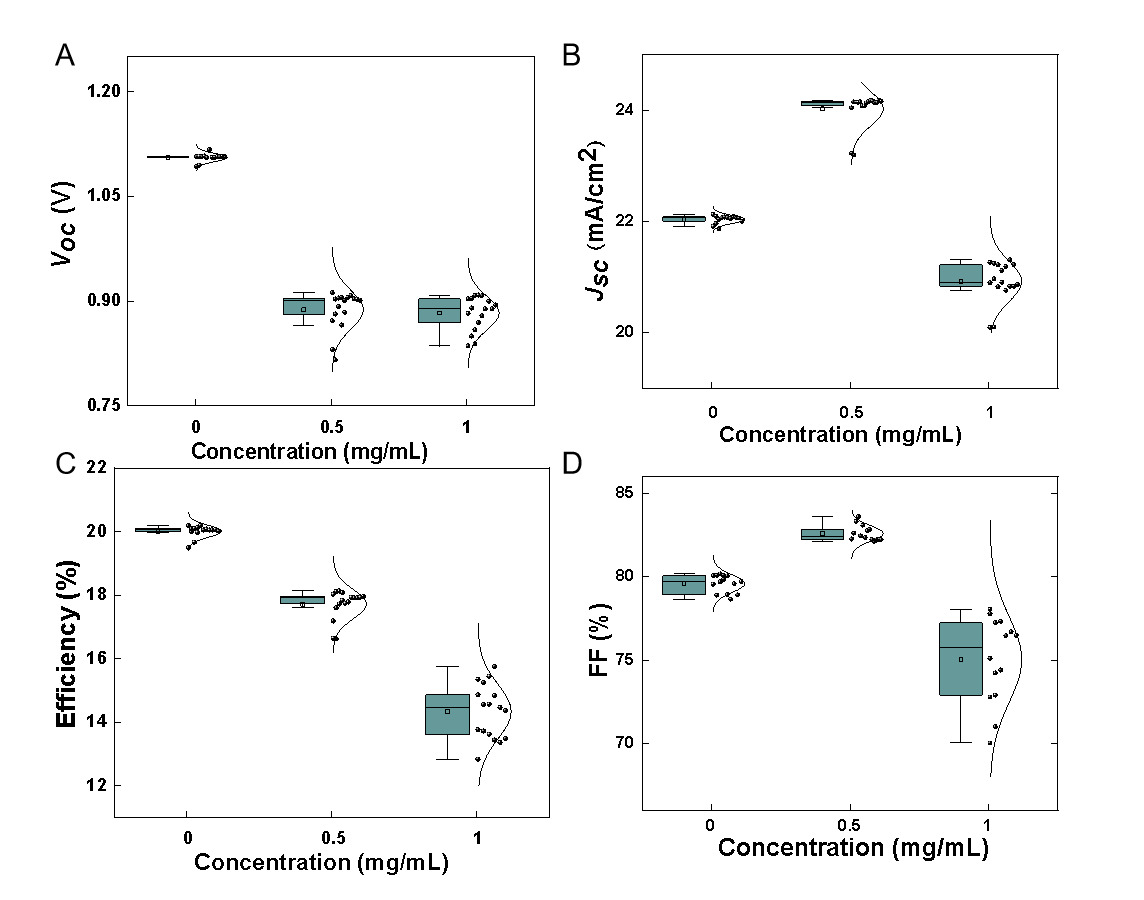


**Fig. S12 |** Statistical distribution of the PCE, *V_oc_*, FF and *J_sc_* of rigid PSCs prepared on the ETLs with different concentration of NDI-C4. (NDI-C4 was mixed with chlorobenzene at different concentrations: 0, 0.5, 1 mg/mL.)


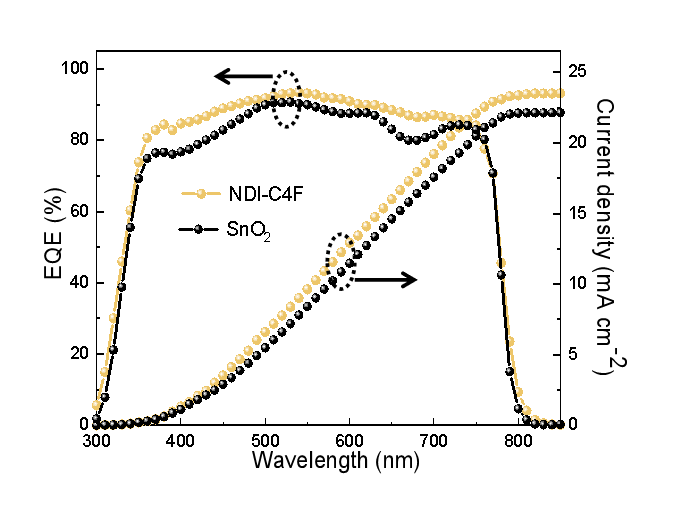


**Fig. S13 |** EQE characterizations of PSCs with SnO_2_ and NDI-C4F.


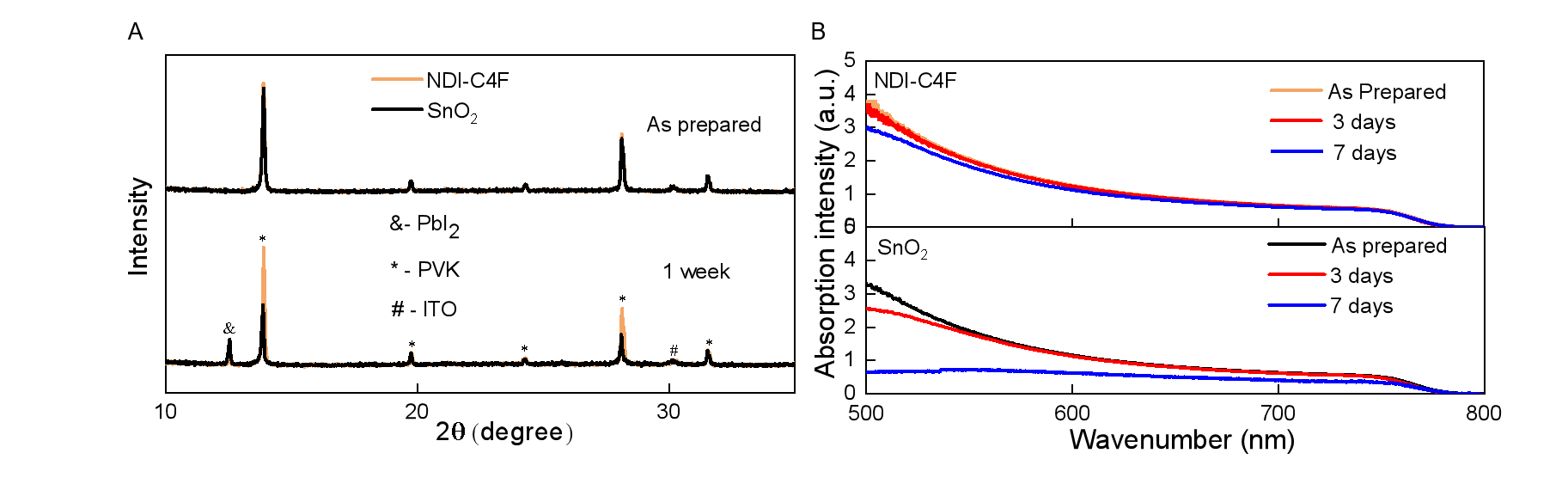


**Fig. S14 |** The change of XRD and UV-vis absorption spectra of perovskite film spin-coated on SnO_2_ and NDI-C4F.

**Supplementary note 2**

**TRPL**

The TRPL decay curves were fitted by using a biexponential decay function:

$$\tau=\frac{A_{1}{\tau_{1}}^{2}+A_{2}{\tau_{2}}^{2}}{A_{1}\tau_{1}+A_{2}\tau_{2}}$$

where *τ*_1_ and *τ*_2_ are the short and long lifetime constants, respectively, while *A*_1_ and *A*_2_ are their corresponding decay amplitudes ^[4]^. As elaborated in the literature, *τ*_1_ is related to the charge transfer to the HTL, whereas *τ*_2_ arises from the interface non-radiative recombination losses ^[5]^.

**Table S1.** The synthesis cost of NDI-C4F and NDI-C4 in lab.

| **Materials** | **Dosage (g)** | **Unit Price ($/kg)** | **Chemical Cost ($)** | **Product ($/g)** |
| --- | --- | --- | --- | --- |
| **NDI-C4F** | | | | |
| 1,4,5,8-naphthalenetetracarboxylic dianhydride | 0.7 | 337.2 | 0.2 | **49.6** |
| 2,2,3,3,4,4,4-heptafluorobutylamine | 1.1 | 34883.7 | 38.4 |  |
| *N*, *N*-dimethylformamide | 23.7 | 2.0 | 0.05 |  |
| Dichloromethane | 861.6 | 2.1 | 1.8 |  |
| MgSO_4_ | 0.8 | 8.8 | 0.07 |  |
| Silica gel | 185.5 | 7.5 | 1.4 |  |
| petroleum ether | 353.6 | 3.3 | 1.2 |  |
| Expect 1.6g  Yield 53% | Product 0.87g |  | 43.1 |  |
| **NDI-C4** | | | | |
| 1,4,5,8-naphthalenetetracarboxylic dianhydride | 2.0 | 337.2 | 0.7 | **3.7** |
| *n*-butylamine | 1.2 | 5.5 | 0.007 |  |
| *N*, *N*-dimethylformamide | 28.3 | 2.05 | 0.07 |  |
| Dichloromethane | 1315.5 | 2.1 | 2.8 |  |
| MgSO_4_ | 1.3 | 8.8 | 0.01 |  |
| Silica gel | 246.8 | 7.5 | 1.8 |  |
| petroleum ether | 288.3 | 3.3 | 0.9 |  |
| Expect 2.8g  Yield 60% | Product 1.68g |  | 6.3 |  |
| **SnO_2_** purchased from Advanced Election Technology Co., LtD | | | | **31.16 $/100mL** |
| **C_60_** purchased from Xi’an Polymer Light Technology Corp (China) | | | | **68.34 $/g** |

**Table S2.** Fitting parameters for time-resolved photoluminescence (TRPL) of perovskite films based on different ETLs on glass substrate.

| Samples | A_1_ (%) | A_2_ (%) | τ_1_ (ns) | τ_2_ (ns) | τ_ave_ (ns) |
| --- | --- | --- | --- | --- | --- |
| ITO/NDI-C4F/perovskite | 25 | 75 | 143.6 | 280.1 | 245.75 |
| ITO/SnO_2_/perovskite | 36 | 64 | 326.7 | 723.5 | 580.08 |

**Table S3.** Fitting parameters for TRPL of perovskite thin bottom films.

| Samples | A_1_ (%) | A_2_ (%) | τ_1_ (ns) | τ_2_ (ns) | τ_ave_ (ns) |
| --- | --- | --- | --- | --- | --- |
| ITO/NDI-C4F/perovskite | 21 | 79 | 225.7 | 520.4 | 458.26 |
| ITO/SnO_2_/perovskite | 20 | 80 | 109.2 | 238.9 | 213.04 |

**Table S4.** The highest PCE and hysteresis index based on rigid, flexible and large area perovskite solar cells devices measured in forward and reverse scan under AM 1.5 G 1 sun illumination of 100 mW cm^2^.

| Devices | | Rigid | | Flexible | | Large area | |
| --- | --- | --- | --- | --- | --- | --- | --- |
|  |  | PCE | HI | PCE | HI | PCE | HI |
| NDI-C4F | Reverse | 23.21% | 3% | 20.40% | 1.4% | 19.09% | 9% |
|  | Forward | 22.46% |  | 20.11% |  | 17.19% |  |
| SnO_2_ | Reverse | 20.33% | 12% | 18.42% | 11.7% | 16.15% | 15.7% |
|  | Forward | 17.89% |  | 16.26% |  | 13.61% |  |

Note: HI is defined as (HI=PCE_reverse_-PCE_forward_)/PCE_reverse_.^[6]^

**Table S5.** Comparison of non-fullerene organic electron transport materials in the literature

for *n-i-p* structure perovskite solar cells.

| Materials | Device structure | PCE(%) | Reference |
| --- | --- | --- | --- |
| NDI-C4F | ITO/NDI-C4F/  Perovskite/Spiro-OMeTAD/Ag | 23.21 | This work  (2023) |
| BTF-BA | ITO/BTF-BA/  Perovskite/Spiro-OMeTAD/Ag | 19.60 | 2022 [7] |
| PDI-LAS | ITO/PDI-LAS/  MAPbI_3_/Spiro-OMeTADAu | 18.77 | 1. [8] |
| NDI-1 | FTO/NDI-1/  FA_0.79_Cs_0.05_MA_0.16_  PbI_2.49_Br_0.51_/SpiroOMeTAD/Au | 14 | 1. [9] |
| PTCDA | ITO/PTCDA/  MAPbI_3_/  PTA /MoO_3_/Ag | 14.3 | (2020) [10] |
| NDI-P | ITO/NDI-P/ MAPbI_3_/  Spiro-OMeTAD/Au | 16 | 1. [11] |
| FPDI | ITO/FPDI/ MA_3_Bi_2_I_9_/Spiro-OMeTAD/Ag | 0.06 | 1. [12] |
| PFN-2TNDI | ITO/PFN-2TNDI /MAPbI_3-x_Cl_x_/  SpiroOMeTAD/Au | 15.96 | 1. [13] |
| CDIN | ITO/PEIE/CDIN  /CH_3_NH_3_PbI_3_/  SpiroOMeTAD/Ag | 17.1 | (2016) [14] |
| N-PDI | FTO/N-PDI/  MAPbI_3-x_Cl_x_ /Spiro-OMeTAD/Au | 17.66 | 1. [15] |

**Table S6.** Summary of parameters obtained from EIS for the devices with different ETLs.

| ETL | R_s_ (Ω) | R_ct_ (Ω) | R_rec_ (Ω) |
| --- | --- | --- | --- |
| NDI-C4F | 20.75 | 10796 | 2.16×10^5^ |
| SnO_2_ | 27.77 | 16154 | 1.49×10^5^ |

**References**

[1] W. Wang, K. Wei, L. Yang, J. Deng, J. Zhang, W. Tang, *Mater. Horiz.* **2023**, *10*, 2609

[2] G. Du, L. Yang, C. Zhang, X. Zhang, N. Rolston, Z. Luo, J. Zhang, *Adv. Energy Mater.* **2022**, *12*, 2103966.

[3] G. Kresse, J. Furthmüller, *Phys. Rev. B,* **1996***,* *54*, 11169.

[4] T. Niu, W. Zhu, Y. Zhang, Q. Xue, X. Jiao, Z. Wang, Y.-M. Xie, P. Li, R. Chen, F. Huang, Y. Li, H.-L. Yip, Y. Cao, *Joule* **2021**, *5*, 249.

[5] Q. Fu, H. Liu, X. Tang, R. Wang, M. Chen, Y. Liu, *ACS Energy Lett.* **2022**, *7*, 1128.

[6] J. Xiong, P. N. Samanta, Y. Qi, T. Demeritte, K. Williams, J. Leszczynski, Q. Dai, *ACS Appl. Mater. Interfaces* **2022**, 14, 5414-5424.

[7] N. Fan, Y. Wang, C. Zhang, G. Zhu, G. Du, K. Wei, J. Deng, Z. Luo, L. Yang, J. Zhang. *J. Mater. Chem. A*, **2022**,10, 8911-8922.

[8] F. Ye, D. Zhang, X. Xu, H. Guo, S. Liu, S. Zhang, Y. Wu and W.-H. Zhu, *Sol. RRL*, **2021**, 5, 2000736.

[9] K. Al Kurdi, D. P. McCarthy, D. P. McMeekin, S. O. Furer, M.-H. Tremblay, S. Barlow, U. Bach and S. R. Marder, *Mater. Chem. Front.*, **2021**, 5, 450-457.

[10] S. Tsarev, S. Y. Luchkin, K. J. Stevenson and P. A. Troshin, Synth. Met., 2020, 268, 116497.

[11] L. Li, Y. Wu, E. Li, C. Shen, H. Zhang, X. Xu, G. Wu, M. Cai and W. H. Zhu, *Chem. Commun. (Camb.)*, **2019**, 55, 13239-13242.

[12] J. Huang, Z. Gu, X. Zhang, G. Wu and H. Chen, *J. Alloys Compd.*, **2018**, 767, 870-876.

[13] D. Li, C.Sun, H. Li, H.Shi, X.Shai, Q.Sun, J. Han, Y.Shen, H. L. Yip,F. Huang and M. Wang, *Chem. Sci.*, **2017**, 8, 4587-4594.

[14] Z. Zhu, J.-Q. Xu, C.-C. Chueh, H. Liu, Z. a. Li, X. Li, H. Chen and A. K. Y. Jen, *Adv. Mater.,* **2016**, 28, 10786-10793.

[15] H. Zhang, L. Xue, J. Han, Y. Q. Fu, Y. Shen, Z. Zhang, Y. Li and M. Wang, *J. Mater. Chem. A*, **2016**, 4, 8724-8733.
